# Dual‐Responsive Supramolecular Polymeric Nanomedicine for Self‐Cascade Amplified Cancer Immunotherapy

**DOI:** 10.1002/advs.202305382

**Published:** 2024-03-17

**Authors:** Wenting Hu, Binglin Ye, Guocan Yu, Huang Yang, Hao Wu, Yuan Ding, Feihe Huang, Weilin Wang, Zhengwei Mao

**Affiliations:** ^1^ Department of Hepatobiliary and Pancreatic Surgery The Second Affiliated Hospital Zhejiang University School of Medicine Hangzhou Zhejiang 310009 China; ^2^ The Second Affiliated Hospital of Zhejiang University Key Laboratory of Precision Diagnosis and Treatment for Hepatobiliary and Pancreatic Tumor of Zhejiang Province Hangzhou Zhejiang 310009 China; ^3^ The Second Affiliated Hospital of Zhejiang University Research Center of Diagnosis and Treatment Technology for Hepatocellular Carcinoma of Zhejiang Province Hangzhou Zhejiang 310009 China; ^4^ The Second Affiliated Hospital of Zhejiang University Clinical Research Center of Hepatobiliary and Pancreatic Diseases of Zhejiang Province Hangzhou Zhejiang 310009 China; ^5^ Clinical Medicine Innovation Center of Precision Diagnosis and Treatment for Hepatobiliary and Pancreatic Disease Zhejiang University Hangzhou Zhejiang 310009 China; ^6^ Cancer Center Zhejiang University Hangzhou Zhejiang 310009 China; ^7^ Key Laboratory of Bioorganic Phosphorus Chemistry & Chemical Biology Department of Chemistry Tsinghua University Beijing 100084 P. R. China; ^8^ MOE Key Laboratory of Macromolecular Synthesis and Functionalization Department of Polymer Science and Engineering Zhejiang University Hangzhou Zhejiang 310027 China; ^9^ Department of Gastroenterology The Second Affiliated Hospital and Yuying Children's Hospital of Wenzhou Medical University Wenzhou Zhejiang 325000 China; ^10^ Stoddart Institute of Molecular Science Department of Chemistry Zhejiang University Hangzhou Zhejiang 310027 China; ^11^ Zhejiang‐Israel Joint Laboratory of Self‐Assembling Functional Materials ZJU‐Hangzhou Global Scientific and Technological Innovation Center Zhejiang University Hangzhou Zhejiang 311215 China

**Keywords:** cancer theranostics, drug delivery systems, host−guest recognition, immunotherapy, supramolecular chemistry

## Abstract

Insufficient tumor immunogenicity and immune escape from tumors remain common problems in all tumor immunotherapies. Recent studies have shown that pyroptosis, a form of programmed cell death that is accompanied by immune checkpoint inhibitors, can induce effective immunogenic cell death and long‐term immune activation. Therapeutic strategies to jointly induce pyroptosis and reverse immunosuppressive tumor microenvironments are promising for cancer immunotherapy. In this regard, a dual‐responsive supramolecular polymeric nanomedicine (NCSNPs) to self‐cascade amplify the benefits of cancer immunotherapy is designed. The NCSNPs are formulated by β‐cyclodextrin coupling nitric oxide (NO) donor, a pyroptosis activator, and NLG919, an indoleamine 2,3‐dioxygenase (IDO) inhibitor, and self‐assembled through host–guest molecular recognition and hydrophobic interaction to obtain nanoparticles. NCSNPs possess excellent tumor accumulation and bioavailability attributed to ingenious supramolecular engineering. The study not only confirms the occurrence of NO‐triggered pyroptosis in tumors for the first time but also reverses the immunosuppressive microenvironment in tumor sites via an IDO inhibitor by enhancing the infiltration of cytotoxic T lymphocytes, to achieve remarkable inhibition of tumor proliferation. Thus, this study provides a novel strategy for cancer immunotherapy.

## Graphical abstract

A supramolecular polymeric nanomedicine is constructed capable of tandem release of the pyroptotic inducer‐NO and the IDO inhibitor‐NLG919 through β‐cyclodextrin/NLG919 host−guest molecular recognition and hydrophobic interaction self‐assembly strategies and GSH/ROS responsive disassembly strategy, enabling self‐cascade amplified dual‐immunotherapy. It enhances therapeutic efficacy and minimizes systemic toxicity.

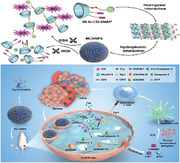

## 1 Introduction

Cancer immunotherapy is an innovative treatment that has revolutionized oncotherapy. The strategy involves attacking cancer cells by reawakening and boosting the immune system.^[^ ^1^, ^2^, ^3^ ^]^ However, only a fraction of patients respond to this treatment, owing to various immune escape mechanisms, such as the immunosuppressive tumor microenvironment (TME), poor immunogenicity, and insufficient intratumoral cytotoxic T lymphocyte (CTL) infiltration, etc.^[^ ^4^, ^5^ ^]^ Current immunotherapies face major barriers. Therefore, the development of a novel and superior nanomedicine with the capacity to sensitize tumor and reverse immune suppression for strengthening antitumor immune responses, is imperative for tumor amplification immunotherapy.

Pyroptosis, known as inflammation‐associated programmed cell death, is characterized by pore formation, cell swelling and bubbling, cell lysis, and the leakage of cell contents containing a large number of inflammatory cytokines.^[^ ^6^, ^7^, ^8^ ^]^ Tumor cells undergoing pyroptosis can induce immunogenic cell death (ICD) by emitting a set of molecules called danger‐associated molecular patterns (DAMPs), which represent an essential immune adjuvant for recruiting and maturating antigen‐presenting cells, provoking a pre‐existing anticancer immune response and enhancing anticancer efficacy.^[^ ^8^, ^9^ ^]^ Recent studies have adopted a pyroptosis‐based mechanism to cure tumors, particularly cancer immunotherapy involving therapeutic regimens including but not limited to chemotherapy,^[^ ^10^ ^]^ photodynamic therapy,^[^ ^11^, ^12^, ^13^ ^]^ and radiotherapy,^[^ ^14^ ^]^ further applications of which have been restrained by drug resistance and severe side effects.

Nitric oxide (NO) is a unique bioactive signaling messenger in diverse physiology and pathophysiology. A recent study confirmed that NO can induce pyroptosis in macrophages.^[^ ^15^ ^]^ However, NO‐based gas therapy has not been reported to trigger pyroptosis in cancer cells. Owing to the advantages of gas therapy, which causes negligible multidrug resistance^[^ ^16^ ^]^and is safe, greener, and efficient, NO‐based pyroptosis therapy can serve as a pyroptosis activator to initiate an immune response for enhancing the immunogenicity of tumors. If the relevant endogenous immunosuppressive pathways can be inhibited simultaneously, it would elicit high expectations of a positive impact in the field of immune oncology by synergizing to revert antitumor immune responses. Indoleamine 2,3‐dioxygenase (IDO), a critical negative feedback enzyme, is involved in the generation of immunosuppressive TMEs.^[^ ^17^ ^]^ IDO mediates its immunosuppressive function by promoting the enzymatic transformation of L‐tryptophan (Trp) into L‐kynurenine (Kyn), resulting in accumulated Kyn providing essential nutrition for rapid tumor growth and degraded Trp interfering with the survival and activity of CTLs.^[^ ^18^ ^]^ Therefore, IDO inhibitor‐mediated immune checkpoint blockade therapy can be employed to reverse immune suppression for strengthening antitumor immune responses.^[^ ^19^, ^20^ ^]^ NLG919, an IDO inhibitor used in the Phase II clinical trial, could stimulate positive feedback from the immune system by regulating the Trp‐Kyn pathway.^[^ ^21^, ^22^, ^23^ ^]^ Thus, NO‐mediated pyroptosis inducers in combination with IDO‐inhibition‐based immunotherapy represent a promising approach for discovering and constructing novel nanomedicines that simultaneously trigger pyroptosis and hamper IDO‐related immune escape, to realize self‐cascade amplification for antitumor immunotherapy.

Nanomedicine has emerged as a promising field in which nanoscale materials and techniques are utilized to revolutionize the diagnosis and treatment of cancer.^[^ ^24^ ^]^ One of the most significant applications of nanomedicine is the targeted delivery of therapeutic agents to the TMEs. TMEs are highly complex and heterogeneous and are characterized by abnormal blood vessel formation, high interstitial pressure, and immunosuppressive features.^[^ ^25^ ^]^ These unique features pose significant challenges to conventional drug delivery methods. Nanomedicine offers innovative solutions by harnessing the distinctive properties of nanoparticles to overcome these barriers and enhance the efficacy of cancer treatments. Numerous nanoscale interventions have been strategically designed to boost cancer immunotherapy.^[^ ^26^, ^27^, ^28^ ^]^ Nanoparticles can be precisely engineered by controlling their size, shape, surface properties, and loading capacity. This enables the design of nanocarriers capable of co‐delivering and specifically releasing NLG919 and S‐nitroso‐N‐acetyl‐DL‐penicillamine (SNAP) into TMEs.

**Figure 1:**
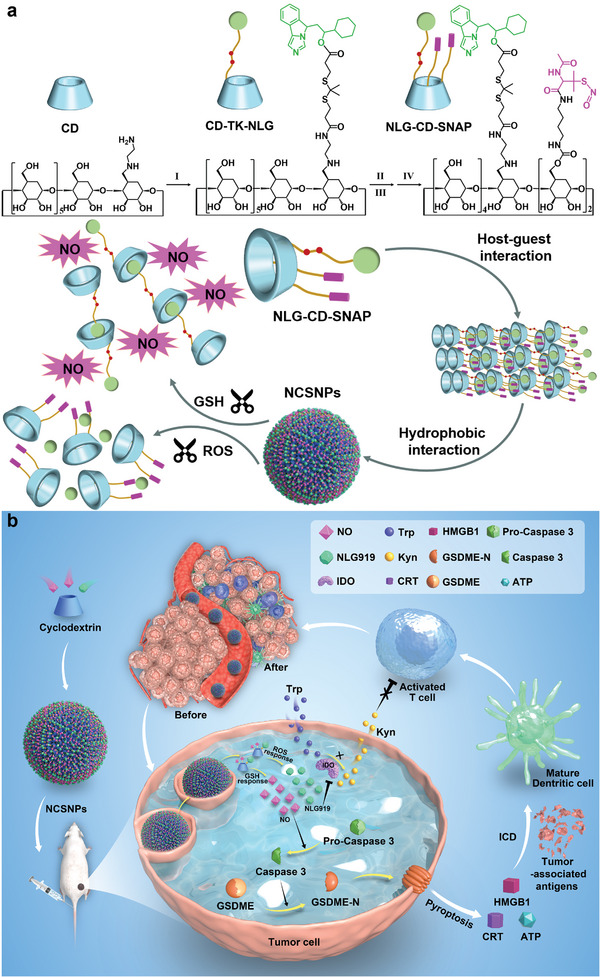
Schematic diagram for dual responsive supramolecular polymeric nanomedicine (NCSNPs), which could be self‐assembled by NLG‐CD‐SNAP through host‐guest recognition and hydrophobic interaction, combinatory immunotherapy by simultaneously conquering tumor immune resistance mechanisms. a) Synthetic routes of NLG‐CD‐SNAP and tumor‐specific activation of NCSNPs. I. NLG‐TK‐COOH, N‐(3‐dimethylaminopropyl)‐*N*′‐ethylcarbodiimide hydrochloride, 4‐(dimethylamino)pyridine, *N*‐hydroxysuccinimide, *N*, *N*‐dimethyl‐formamide; II. 1,1′‐carbonyldiimidazole, *N*, *N*‐dimethylformamide; III. DAB‐SH, triethylamine, *N*, *N*‐dimethylformamide; IV. tert‐butylnitrite, *N*, *N*‐dimethylformamide. b) Schematic illustration of the processes involved in dual‐responsive immune‐modulatable NCSNPs preparation, in vivo delivery, and the cascade‐amplified combination of pyroptosis and immune checkpoint inhibitors. Upon intravenous injection, NCSNPs could passively accumulate in the tumor through enhanced permeation and retention effect, and rapidly release the loaded NO and NLG under the reaction of tumor reactive oxygen species (ROS) and glutathione (GSH). Such NO could trigger strong pyroptosis of cancer cells, characterized by the enhanced release of DAMPs such as calreticulin (CRT), high mobility group box 1 (HMGB1), and ATP for alerting the host's immune system. Consequently, specific antitumor immunity is initiated to promote the maturation of dendritic cells (DCs), intratumoral infiltration of effector T cells and secretion of proinflammatory cytokine interferon‐gamma (IFN‐γ). In the meantime, tumor‐accumulated NLG919 could block the IDO1‐mediated immunosuppressive pathway. As a result, NCSNPs elicit a profound antitumor immune response with NO‐triggered pyroptosis and combat IDO‐1‐inducible adaptive immune resistance with NLG.

Herein, we designed a supramolecular nanomedicine that adopted a strategy comprising molecular self‐assembly and GSH/ROS cascade‐responsive disassembly for tandem dual‐immunotherapy‐inflammation‐related pyroptosis and blocking IDO‐mediated immunosuppression (**Figure** **1**). Such supramolecular self‐assembly via non‐covalent interactions has incomparable advantages in cancer immunotherapy because of its dynamic properties and stimuli‐responsive behaviors.^[^ ^29^, ^30^, ^31^, ^32^, ^33^, ^34^, ^35^ ^]^ A cyclodextrin (CD)‐based supramolecular self‐assembly system, in which CDs functioned as the primary component of the green scaffold to co‐deliver therapeutic agents (NLG and NO generator), was selected to fabricate this nanomedicine. NLG and NO donors were conjugated with CD via a thioketal moiety as the ROS‐trigger linker and an S‐nitrosothiol bond (‐SNO) as the GSH‐trigger linker, and the resulting monomer was denoted as NLG‐CD‐SNAP. Through microfluidic technology, NLG‐CD‐SNAP could self‐assemble into a polymeric nanomedicine (denoted as NCSNPs) via host‐guest recognition and hydrophobic interactions, serving as a drug reservoir for controlled release of the loaded cargoes in the tumors. After entering the TME, NLG and NO were sequentially discharged through GSH/ROS‐mediated disassembly. NO release caused pyroptosis, which could release immune‐stimulatory cytokines and recover the strong inflammatory effect; while NLG was activated to cut off the IDO pathway, which could reverse the immunosuppressive environment. The combination of pyroptosis inducers (NO) and immune checkpoint blockade drugs (NLG) exhibited improved antitumor performance. Thus, the outcomes of this strategy would pave a new way for cancer immunotherapies.

## 2 Results and Discussion

**Figure 2:**
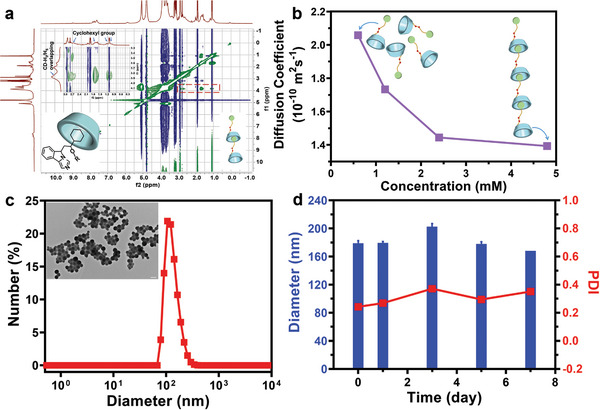
a) 2D NOESY spectrum (400 MHz, room temperature) of CD‐TK‐NLG. b) Concentration dependence of the diffusion coefficient for CD‐TK‐NLG (from ^1^H NMR spectroscopy, 500 MHz, D_2_O, 300 K). c) Hydrodynamic diameter distribution obtained for NCSNPs. The inset is the corresponding NCSNPs TEM image. The scale bar is 200 nm. d) The stability of NCSNPs in aqueous solution for 7 days (*n* = 3).

The supramolecular monomer (NLG‐CD‐SNAP) was first synthesized via a multistep synthetic route with high yield and purity (Scheme S1 and Figures S1–S15, Supporting Information). The synthesis of the designed monomer started with the esterification reaction to obtain NLG‐TK‐COOH with a terminal carboxyl group and a thioketal linker. Then, it was conjugated to β‐CD‐NH_2_, and the obtained CD‐TK‐NLG was activated by 1,1′‐carbonyldiimidazole and reacted with the amine group on DAB‐SH to give a monomer in thiol form. The nitrosation reaction in the presence of tert‐butyl nitrite yielded the final supramolecular monomer. Additionally, SNAP‐CD‐Fc was synthesized via the conjugation of NO donors to β‐CD and was fully characterized for a follow‐up comparison experiment (Scheme S2 and Figure S16, Supporting Information). Ce6‐NLG‐CD‐SNAP was obtained through the esterification reaction between the carboxyl groups of Ce6 (a fluorescent group containing three carboxyl groups) and the hydroxyl group of NLG‐CD‐SNAP, and was fully characterized for a follow‐up cell experiment (Scheme S3 and Figure S17, Supporting Information). Since the maximum internal diameter of the β‐CD cavity is ≈7.90 Å,^[^ ^36^, ^37^ ^]^ the NLG molecule could easily be embedded in the nonpolar interior of the β‐CD cavity.^[^ ^38^ ^]^ The result of the inclusion complexation between NLG and β‐CD was validated by NMR‐NOESY experiments (**Figure** **2**a; Figure S18, Supporting Information). Indeed, strong correlations were observed between the NLG protons (cyclohexyl group) and H‐3 and H‐5 protons inside the CD cavity. Such correlations were consistent with the intermolecular inclusion of the NLG group across the secondary rim of the β‐CD of another monomer. The self‐assembled structure of CD‐TK‐NLG was confirmed by NMR‐DOSY spectroscopy and the diffusion coefficient (*D*) was plotted as a function of the concentration (*C*) of each monomer. The diffusion coefficient of CD‐TK‐NLG showed a nonlinear decrease with an increase in its concentration and was smaller than that of the native β‐CD (D = 2.60 × 10^−10^ m^2^·s^−1^) over the same concentration range,^[^ ^39^ ^]^ confirming the polymerization of the repeat unit in the aqueous solution (Figure 2b; Figure S18, Supporting Information). The results gave direct evidence of host‐guest self‐assembly in the aqueous solution. In addition, to guarantee the utility of pyroptosis activators of NO and maintain good solubility of the self‐assembly in a physiological environment, the conjugation of β‐CD with different ratios of NO donors was investigated. Theoretically, the number of NO donors should be maximized. In the synthesis process, the feeding ratio of NO donors was more than four equivalents, and the solubility of the obtained supramolecular monomer in water plummeted, directly affecting its subsequent use under physiological conditions. A supramolecular monomer containing two NO donors was eventually identified. Another supramolecular monomer SNAP‐CD‐Fc used in the control experiment had the same number of NO donors.

After the formation of a potent host‐guest complex was confirmed, dynamic light scattering (DLS) and transmission electron microscopy (TEM) were performed to assess the size distribution and morphology of the obtained self‐assemblies, which was denoted as NCSNPs. The NCSNPs possessed an average diameter of 179 ± 4.3 nm (polydispersity index, PDI = 0.243 ± 0.04) and zeta potential of +11.5 ± 0.4 mV, which is in the range for potential in vivo application (Figure 2c,d; Figure S19, Supporting Information). The formation of a regular and well‐defined spherical object was detected by TEM (Figure 2c). Considering the influence of the liquid layer on top of the particles, the so‐called hydrodynamic diameter obtained via DLS typically exceeds the size given in the dry state under TEM. Moreover, the DLS results in water, saline, and fetal bovine serum showed little change over 7 days, indicating great stability of the NCSNPs (Figure 2d; Figure S20, Supporting Information). The control groups (denoted as NCNPs and SCNPs) self‐assembled from CD‐TK‐NLG and SNAP‐CD‐Fc, respectively, presented slightly different sizes than NCSNPs (Figures S21 and S22, Supporting Information).

**Figure 3:**
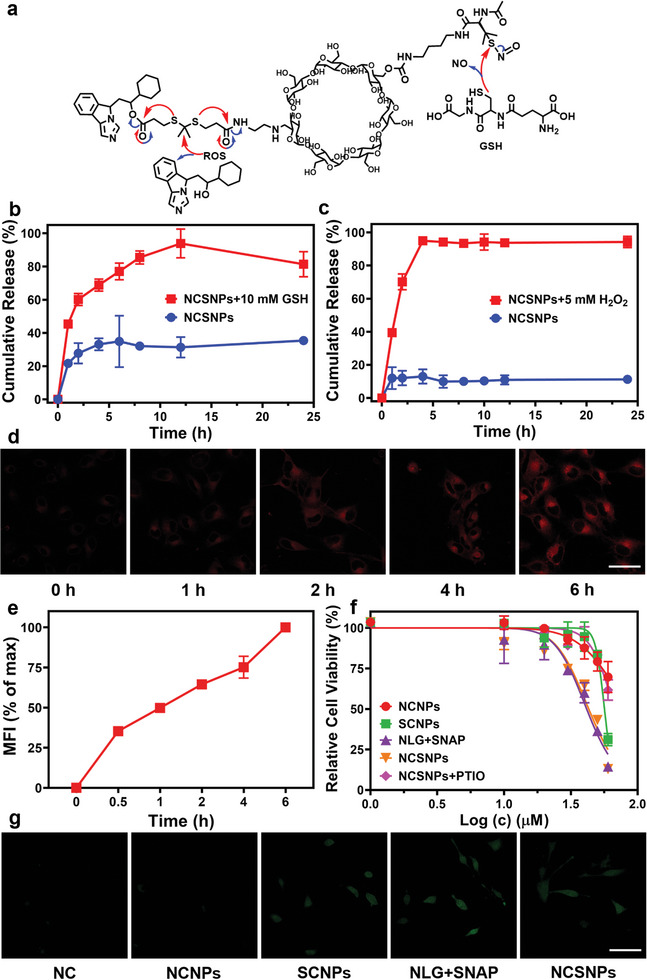
a) Scheme illustration of the GSH/ROS dual‐responsive release of NLG prodrug and NO donor from NCSNPs. b) Release of NO donor from NCSNPs in the presence and absence of GSH. c) Release of NLG prodrug from NCSNPs in the presence and absence of H_2_O_2_. d) Cellular uptake of NCSNPs after 0, 1, 2, 4, and 6 h of incubation. Ce6‐labeled NCSNPs (red) were used to visualize the process of their cellular internalization. The scale bar is 20 µm. e) Mean fluorescence intensity (MFI) of NCSNPs at varying periods. f) Viability curves of B16 cells after treatment with NCNPs, SCNPs, NLG+SNAP, NCSNPs, NCSNPs + PTIO for 24 h. The concentration of Carboxy‐PTIO in inset (f) is 20 µM. g) CLSM images of intracellular NO (DAF‐FM, green) generation in B16 cells under different treatments. The scale bar is 20 µm.

To further improve the selective delivery of anticancer drugs, GSH/ROS dual‐responsive NCSNPs were designed with the expectation of the precise release of the pyroptotic activator NO and the IDO inhibitor NLG at the tumor site to achieve effective cancer therapy (**Figure** **3**a). As one of the unique hallmarks of cancer, ROS offer high selectivity because the ROS concentration in normal cells is relatively low (10–700 nm). There is substantial evidence that high levels of ROS production in tumor cells mainly depend on mitochondrial dysfunction. GSH, a reduced biothiol, is considered the main redox buffer to preserve cell survival under oxidative stress. High ROS levels are often accompanied by high GSH levels in tumors.^[^ ^40^ ^]^ The GSH level in tumor cells can reach 2–10 mm, which is far higher than that of normal cells (2–20 µm).^[^ ^29^, ^34^, ^41^, ^42^, ^43^ ^]^ Compared with nanomedicines simply responding to ROS or GSH, the redox dual‐triggered strategy presents a faster response rate and higher efficiency of therapeutic reagent release.^[^ ^44^, ^45^, ^46^, ^47^, ^48^ ^]^ The NO release behavior was evaluated by incubating NCSNPs in different concentrations of a GSH solution and measured by the Griess reagent kit. A high level of intracellular GSH could be an efficient catalyst for the decomposition of S‐nitrosothiols (RSNOs) into NO and the corresponding disulfides (RSSR).^[^ ^49^ ^]^ As shown in Figure 3b, upon incubation in 10 mm GSH for 4 h, more than 68.9% of the NO was released from the NCSNPs, whereas a small amount of NO was detected in the physiological environment. The thioketal linker in the NCSNPs was ROS‐sensitive, which could be oxidized by H_2_O_2_ to release active NLG. As shown in Figure 3c, NLG could not be released from the NCSNPs under a physiological environment, and less than 11.3% of the loaded NLG leaked out from the nanoplatform. In contrast, the release of NLG was dramatically accelerated upon incubation in the presence of H_2_O_2_, and 94.3% of the free NLG was released after 24 h. Moreover, the release behavior of NO and NLG from NCSNPs was investigated in 10 mm GSH and 5 mm H_2_O_2_ solutions. The release profiles (Figure S24, Supporting Information) indicated that ≈91.2% NO and 94.5% NLG were released after 8 h. Meanwhile, the TEM results showed that NCSNPs collapsed rapidly upon incubation with a dual‐responsive environment, in sharp contrast to the physiological environment, confirming the decomposition of NCSNPs and the release of the two therapeutic reagents (Figure S25, Supporting Information). These results suggested that NCSNPs could be broken down at high concentrations of GSH/ROS, leading to payload release. Therefore, NCSNPs are expected to maintain stability during blood circulation and achieve explosive drug release after internalization by tumor cells, which is conducive to reducing side effects on normal tissues.

Endocytosis of nanoparticles into cells is a prerequisite for their efficacy. Based on this, confocallaser scanning microscopy (CLSM) was used to monitor the intracellular internalization of NCSNPs (Figure 3d). Ce6‐labeled NCSNPs were prepared by coupling reaction of e6 chloride (Ce6), a fluorescent group containing three carboxyl groups, with NCSNPs. The fluorescence intensity of the Ce6‐labeled NCSNPs (red) increased with incubation time. The NCSNPs successfully illuminated B16 cells in a time‐dependent manner and exhibited rapid uptake into the cells within 6 h (Figure 3e). This suggested that NCSNPs could be effectively internalized by tumor cells. The cytotoxicity of NCSNPs was then investigated in B16 tumor cells using CCK8 assay. The NCSNPs showed cytotoxicity comparable to that of the two free drugs (NLG + SNAP); meanwhile, the NO scavenger (carboxy‐PTIO) could counteract the cytotoxicity induced by the NCSNPs (Figure 3f; Figure S26, Supporting Information). These observations revealed that the NO and NLG components of the NCSNPs were mainly responsible for the reduced proliferation of B16 cells. Furthermore, we selected human umbilical vein endothelial cells (HUVECs) to investigate the dose‐dependent effects of NCSNPs. As expected, following incubation with three nanomedicines at different concentrations (10–60 µm), the HUVECs exhibited close to normal cell viability, indicating that the nanomedicines were safe for normal cells (Figure S27, Supporting Information). The generation of intracellular NO was traced using the activatable probe 3‐amino‐4‐aminomethyl‐2′, 7′‐difluorescein, diacetate (DAF‐FMDA), whose fluorescence was measured after it was oxidized into the corresponding benzotriazole derivative (Figure 3g). Quantitative measurements indicated that NO‐based treatments (SCNPs, NLG + SNAP, and NCSNPs) significantly increased intracellular NO levels, whereas the NLG component had almost no effect on NO release. Thus, the efficient release of NO was demonstrated in an intracellular reductive environment (Figures S28 and S29, Supporting Information).

The outstanding performance on endocytosis and cytotoxicity of the NCSNPs motivated us to investigate whether they could function as a pyroptosis‐mediated immunotherapy nanoplatform. The appropriate concentrations of NLG and SNAP were explored in further in vitro experiments. NLG is considered to block IDO‐mediated immunosuppressive pathways and SNAP should promote robust tumor regression. The cytotoxicity and IDO‐1 inhibitory activity of NLG were evaluated to determine the optimal therapeutic concentration for maximizing the therapeutic efficacy. It is worth noting that NLG, which has been reported as a nontoxic drug, showed obvious cytotoxicity when the concentration exceeded 100 µm (Figure S30, Supporting Information). At a concentration range of 10 to 100 µm, it had a strong inhibitory effect on IDO enzyme activity (Figure S31, Supporting Information). SNAP suppressed the growth of B16 tumor cells in a dose‐dependent manner. Considering the certain and precise NLG/SNAP ratio (1:2) in the NCSNP chemical structure, the therapeutic formulation with an NLG concentration of 50 µm and a SNAP concentration of 100 µm was preliminarily selected to study both the IDO inhibitory activity and the tumor growth inhibitory capacity.

**Figure 4:**
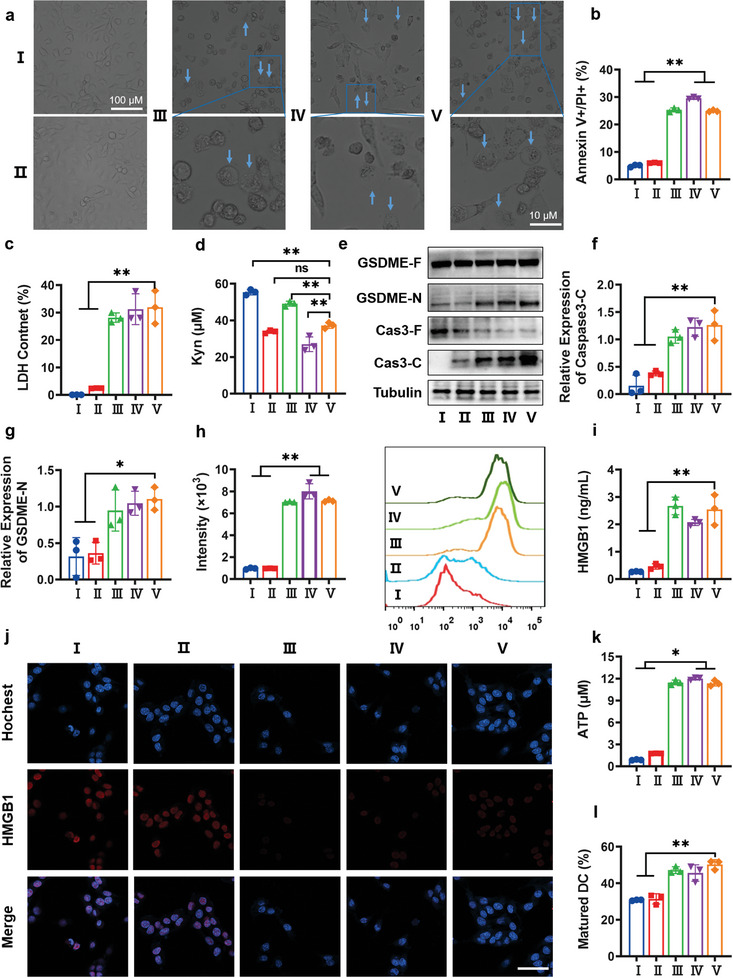
a) Observation of B16 cell morphology after different treatments. The blue arrows and the close‐ups indicate the cells showing a pyroptotic morphology. The scale bar is 100 or 10 µm. b) Annexin‐V/PI assay of B16 cells after different treatments. c) Leakage of intracellular content of the LDH in B16 cells after various treatments. d) Quantitative analysis of Kyn on IDO pathway after various treatments. e) Detection of the pyroptosis‐related protein in B16 cells by Western blot after different treatments. f) Detection of caspase‐3 activation in B16 cells after different treatments. g) Detection of GSDME‐N terminal expression in B16 cells after different treatments. h) Flow cytometry analysis of CRT expression on the B16 cells after different treatments and their mean fluorescence intensity. i) Quantitative detection of supernatant HMGB1 released from the nucleus of the B16 cells after different treatments. j) CLSM images showing the nucleus HMGB1 from B16 cells after different treatments. Blue fluorescence shows nucleic; red fluorescence comes from HMGB1. The scale bar is 100 µm. k) ATP levels in the supernatant of B16 cells after different treatments. l) The percentage of matured DCs after co‐incubation with B16 cells received various treatments. Data are presented as means ± S.D (n = 3). (^***^ *p* < 0.001, ^**^ *p* < 0.01, ^*^ *p* < 0.05). I: control; II: NCNPs; III: SCNPs; IV: NLG+SNAP; V: NCSNPs.

First, the morphological changes of cells after different pre‐treatments were examined via light microscopy (**Figure** **4**a). Materials containing NO components (including but not limited to nanoparticles) exhibited characteristic morphological changes of the pyroptotic cell‐that is, a large amount of cells swelled and bubbled (the blue arrows indicate the sites), compared with NCNPs and the negative control after 24 h of treatment. These results indicated that NO could remarkably induce pyroptosis. Annexin V‐FITC and propidium iodide (PI) dual‐staining was performed to estimate the approximate proportion of cells undergoing pyroptosis. Red fluorescence in the nucleus and green fluorescence in the membrane were observed after incubation with NCSNPs (Figure S32, Supporting Information), indicating that the NCSNP‐mediated nanoplatform triggered pyroptosis, and the ratio of pyroptotic cells was calculated to be ≈24.9% (Figure 4b). Because pyroptosis is accompanied by cell content leakage, we investigated the extracellular release of lactate dehydrogenase (LDH). The amount of released LDH was remarkably larger for NCSNPs and other groups containing NO components in the pre‐treated cells than that for NCNPs and the negative control, confirming the excellent performance of NO‐mediated pyroptosis (Figure 4c). The inhibitory effect of the NCSNPs on the IDO pathway was evaluated in B16 tumor cells. IDO is an interferon‐gamma (IFN‐γ) inducible enzyme that can catalyze the breakdown of Trp into Kyn, where the intratumoral accumulation of Kyn impairs the proliferation of CTLs. The results indicated that the NCSNPs displayed good IDO inhibitory activity, whereas the concentration of Kyn was calculated to be ≈37.2 µm, which is comparable to that of NCNPs (34.7 µm) and slightly lower than that of small molecule drugs (Figure 4d). This suggested that the inhibitory capacity of NLG would not disturbed when encapsulated into NCSNPs and probably ascribed to efficient internalization and ROS‐responsive release.

To clarify the mechanism of NCSNP‐induced pyroptosis, the expression levels of pyroptosis‐related proteins were tracked via Western blot analysis (Figure 4e). The essential pyroptotic protein GSDME induced cell rupture via pore‐formation on the cell membrane. Activated caspase‐3 cleaved GSDME into an N‐terminal fragment of GSDME (GSDME‐N), which could translocate into the plasma membrane and induce a pore‐forming capacity and subsequent release of cell contents, resulting in a pyroptosis‐related immune response.^[^ ^50^ ^]^ Compared with the control group, the cleaved caspase‐3 and GSDME‐N levels in the NCNPs exhibited no noticeable change. NCSNPs treatment significantly stimulated the expression of cleaved caspase‐3 (Figure 4f) and GSDME‐N (Figure 4g). These results confirmed that NCSNPs could induce pyroptosis in vitro.

Pyroptosis has been proven to be a form of ICD,^[^ ^51^ ^]^ which promotes the expression of calreticulin (CRT) on the cell surface, the transposition of high mobility group box 1(HMGB1) outward the nucleus, and ATP release. All these signaling molecules, known as DAMPs, can bind to pattern recognition receptors on dendritic cells (DCs), recruit DCs into tumors, promote DC maturation, and activate cytotoxic T cells. Therefore, the release of DAMPs and the maturation of DCs were quantitatively measured in vitro. The CRT exposed on the cell surface would provide “eat me” signals to macrophages and immature DCs to ingest the dying tumor cells.^[^ ^52^ ^]^ CRT expression was measured using flow cytometry after staining with a fluorochrome‐conjugated CRT antibody. The amount of CRT on the cell membrane treated with NLG + SNAP, SCNPs, and NCSNPs was roughly 7.44‐fold higher than that with the negative control and NCNPs‐treated cells (Figure 4h). Extracellular HMGB1 ignites the secretion of cytokines and chemokines by immune cells, thereby maintaining and prolonging the inflammatory immune microenvironment.^[^ ^53^ ^]^ Enzyme‐linked immunosorbent assay (ELISA) and immunofluorescence were employed to evaluate the transposition of HMGB1 on account of NCSNPs‐triggered ICD (Figure 4i,j). The cell incubation with NCSNPs could significantly stimulate HMGB1 leaking out of the cells, which was comparable to that with SCNPs and NLG + SNAP, whereas minimal changes in HMGB1 expression were detected from NCNPs‐treated cells. Concomitantly, a significant increase in ATP release was observed in cells after treatment with NCSNPs (Figure 4k). Additionally, DC maturation is also an important feature of ICD. After co‐culturing pyroptotic cancer cells with immature DCs, the expression of costimulatory molecules including CD80 and CD86 was measured to assess DC maturation. The percentage of mature DCs markedly increased to 50.3% when the cells were treated with NCSNPs (Figure 4l; Figure S33, Supporting Information), indicating that NCSNPs triggered the maturity of DCs. All evidence indicated that this nanoplatform had the potential to trigger pyroptosis and promote ICD‐mediated immunity, thereby enhancing therapeutic efficacy.

**Figure 5:**
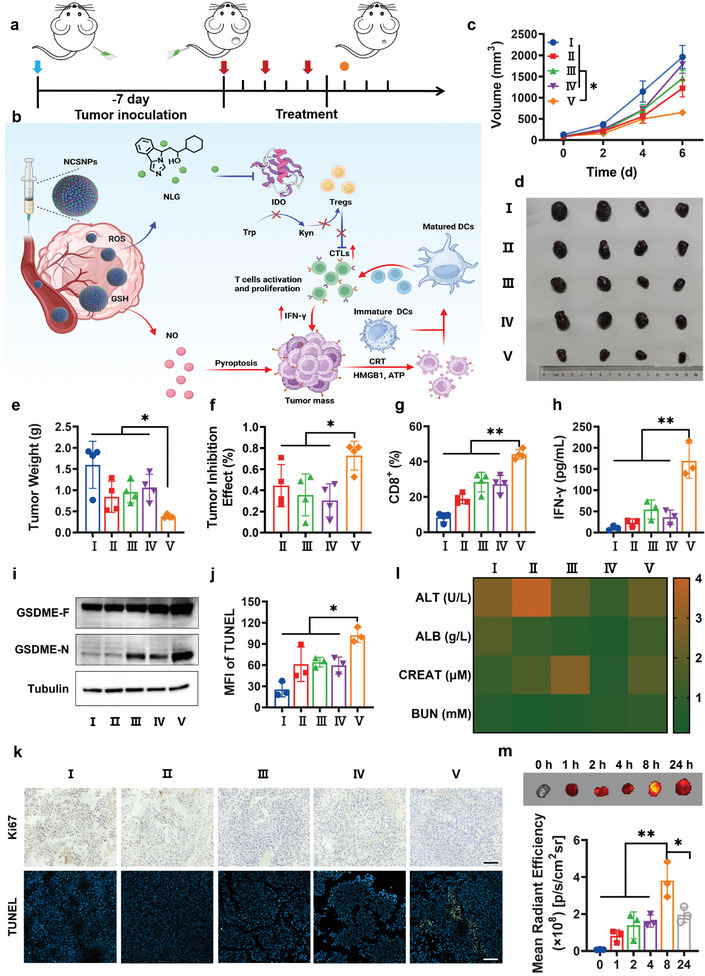
a) Therapeutic schedule for NCSNPs‐mediated tumor immunotherapy. b) Schematic illustration of NCSNPs for effective cancer immunotherapy‐pyroptosis induced by NO and the IDO‐mediated immunosuppression interfered with NLG. c) The tumor volume curves within the treatment period. d) The photographs of the dissected tumors by the end of treatment. e) The final tumor weight. f) one “asterisk” result between group V and others. g) The quantitative analysis of the intratumoral infiltration of CD8^+^ T cells in the tumor. h) IFN‐γ secretion in sera detected by ELISA after different treatments. i) Detection of the pyroptosis‐related protein by Western blot in the tumor. j) MFI of TUNEL positive cells ratio. k) Ki67 and TUNEL staining of tumor sections collected from mice intravenously injected with different treatments. The scale bar is 100 µm. l) Quantitative analysis of liver function biomarkers (ALT and ALB) and kidney function biomarkers (BUN and CREA) in the blood of the mice after various treatments. m) In vivo time‐dependent the tumor site fluorescence intensity and image of B16 tumor‐bearing mice after intravenous injection of NCSNPs. Data are presented as means ± S.D (*n* = 3–5). (^***^ *p* < 0.001, ^**^ *p* < 0.01, ^*^ *p* < 0.05). I: saline; II: NCNPs; III: SCNPs; IV: NLG+SNAP; V: NCSNPs.

Encouraged by their outstanding in vitro performance, we evaluated the antitumor performance of the NCSNPs in B16‐tumor‐bearing mice (**Figure** **5**a). The nanodrugs (NCNPs, SCNPs, and NCSNPs) or small molecule drugs (NLG + SNAP) were intravenously injected into mice every 2 days (equivalent NLG dose of 20 mg·kg^−1^, equivalent SNAP dose of 31.2 mg·kg^−1^). The mice administrated with NCSNPs exhibited obvious therapeutic effects (Figure 5c–e; Figure S34, Supporting Information). In mice treated with the NLG + SNAP group, minimal antitumor capacity was observed, which was comparable to that of the saline group. This was probably due to the poor water solubility of NLG and the poor stability of SNAP. Administration of NCSNPs strongly inhibited tumor growth. The average tumor weight of the NCSNPs group was only 0.38 ± 0.04 g by the end of the treatment, which was far smaller than those of the NCNPs and SCNPs groups, confirming the self‐cascade amplification characteristics of the nanoplatform. The tumor inhibition ratios of NCNPs, SCNPs, NLG + SNAP, and NCSNPs were 44.5%, 35.7%, 30.5%, and 72.9%, respectively (Figure 5f). CTLs (CD3^+^CD8^+^) are the preferred immune cells for killing target cancer cells, and helper T cells (CD3^+^CD4^+^) play an important role in regulating the adaptive immune system. To assess the influence of the intratumoral infiltration of T lymphocytes on the therapeutic outcome, intratumoral CD4^+^ T and CD8^+^ T cells were evaluated using flow cytometry (Figure S35, Supporting Information). Quantitative analyses indicated that the infiltration of CD8^+^ T cells was significantly upregulated to 44.2% in the mice treated with NCSNPs, which was 4.89‐, 2.33‐, 1.56‐, and 1.63‐fold higher than those for saline, NCNPs, SCNPs, and NLG + SNAP, respectively (Figure 5g; Figures S36 and S37, Supporting Information). This implied that NCSNPs could significantly stimulate the antitumor immune response through pyroptosis and IDO‐inhibition. Furthermore, IFN‐γ, secreted by cytotoxic T cells, is another key moderator involved in the activation of cellular immune response.^[^ ^23^ ^]^ The concentration of IFN‐γ secretion in sera was quantitatively measured to be 10.8, 22.8, 54.0, 36.0, and 169 pg·mL^−1^ after treatment with saline, NCNPs, SCNPs, NLG + SNAP, and NCSNPs, respectively (Figure 5h). The IFN‐γ level was tremendously increased after treatment with NCSNPs, indicating a strong immune response through the NCSNPs‐mediated nanoplatform. Meanwhile, in an inflammatory environment, IFN‐γ not only functions as a cytotoxic cytokine to establish a positive feedback loop but also enables the upregulation of the IDO pathway, thereby stimulating other immune‐suppressive mechanisms.^[^ ^54^ ^]^ The employment of IDO inhibitors can substantially inhibit this opposite effect. The Kyn content was calculated to assess the IDO activity. The amount of Kyn was significantly lower in the tumor site in mice treated with NCSNPs (Figure S38, Supporting Information), which is important for immune response stimulation. To confirm the pyroptotic effect of NCSNPs in vivo, Western blotting was employed to detect the expression of GSDME‐N terminal in tumor tissues. A high level of GSDME‐N expression was observed in the tumor site in mice treated with SCNPs and NCSNPs among these groups, indicating that the NO section of NCSNPs successfully induced pyroptosis (Figure 5i). These results confirmed that the high antitumor efficacy benefited from the self‐cascade of NO‐initiated pyroptosis and NLG‐mediated IDO inhibition. This satisfactory antitumor efficacy was supported by histological analyses, including Ki67 staining and transferase‐mediated dUTP nick endlabeling (TUNEL) staining (Figure 5k). According to the TUNEL assay, the most severe damage to tumor cells occurred in the NCSNPs group, with a 4.03‐fold higher mean fluorescence intensity (MFI) compared with the control group (Figure 5j; Figure S39, Supporting Information).

The short circulation time and rapid clearance limit the efficacy of small molecule drugs, whereas nanoprodrugs benefit from the enhanced permeation and retention effect, markedly extending the retention time and boosting tumor accumulation. Using IR780 as a NIR fluorescence probe, fluorescence imaging was employed to monitor the delivery of IR780‐labelled NCSNPs in vivo (Figure 5m). The fluorescence signal indicated that the accumulation of NCSNPs@IR780 in the tumor site occurred quickly, which was observed 1 h post‐injection and could still be traced even 24 h post‐injection, providing strong evidence for the high tumor accumulation rate of NCSNPs. Semi‐quantitative analysis indicated time‐dependent delivery in the tumor site, which enhanced the therapeutic effect. Even with high‐dose injections, the body weights (Figure S40, Supporting Information), major organs (Figure S41, Supporting Information) and key indices of hepatotoxicity and nephrotoxicity from the blood (Figure 5l) of mice treated with NCSNPs did not change, indicating the biosafety and biocompatibility of NCSNPs.

## 3 Conclusion

In summary, we constructed a supramolecular self‐assembly nanoplatform based on amphiphilic self‐assembly and β‐CD mediated host‐guest molecular recognition for the self‐cascade amplifying effect of inflammation‐related pyroptosis with the help of blocking the IDO‐mediated immunosuppression. Attributing to the supramolecular chemistry and nanotechnology, the NO donor and NLG919 were co‐delivered and significantly accumulated in the tumor sections. GSH‐triggered rapid release of NO and ROS‐induced leakage of NLG919 were achieved after cellular internalization through the TME. The released NO functioned as a pyroptosis initiator to stimulate inflammatory cytokine secretion; simultaneously, the activated NLG functioned as an immune checkpoint inhibitor to recover antitumor immune responses. NCSNPs exhibited high antitumor activity and low systemic toxicity in vitro and in vivo experiments. To the best of our knowledge, there are no reports on the preparation of nanoparticles using a combination of NO and NLG to assess their therapeutic efficacy. In this study, we employed a supramolecular self‐assembly system to explore a self‐cascading strategy for amplifying the therapeutic effects.

## Conflict of Interest

The authors declare no conflict of interest.

## Supporting information

Supporting Information

## Data Availability

The data that support the findings of this study are available from the corresponding author upon reasonable request.
