# Supplementary material for: Dual‐Responsive Supramolecular Polymeric Nanomedicine for Self‐Cascade Amplified Cancer Immunotherapy
Source: Adv Sci (Weinh). 2024 Mar 17;11(20):2305382. doi: 10.1002/advs.202305382 (PMC11132052; doi:10.1002/advs.202305382)
Supplement: Supplementary file 1 — Supporting Information [file ADVS-11-2305382-s001.pdf]

ADVANCED SCIENCE
Open Access

Supporting Information

for *Adv. Sci.*, DOI 10.1002/advs.202305382

Dual-Responsive Supramolecular Polymeric Nanomedicine for Self-Cascade Amplified Cancer Immunotherapy

Wenting Hu, Binglin Ye, Guocan Yu, Huang Yang, Hao Wu, Yuan Ding, Feihe Huang, Weilin Wang and Zhengwei Mao**

Dual-Responsive Supramolecular Polymeric Nanomedicine for Self-Cascade Amplified Cancer Immunotherapy

Wenting Hu†, *Binglin Ye*†, *GuocanYu*, *Huang Yang*, Hao Wu, *Yuan Ding*, *Feihe Huang*, *Weilin Wang**, *Zhengwei Mao**

Dr. W. Hu, B. Ye, Dr. Y. Ding, Prof. Dr. W. Wang, Prof. Dr. Z. Mao
Department of Hepatobiliary and Pancreatic Surgery, The Second Affiliated Hospital, Zhejiang University School of Medicine Hangzhou, Zhejiang 310009, China
E-mail: wam@zju.edu.cn
Dr. W. Hu, B. Ye, Dr. Y. Ding, Prof. Dr. W. Wang, Prof. Dr. Z. Mao
The Second Affiliated Hospital of Zhejiang University, Key Laboratory of Precision Diagnosis and Treatment for Hepatobiliary and Pancreatic Tumor of Zhejiang Province, Hangzhou, Zhejiang 310009, China
Dr. W. Hu, B. Ye, Dr. Y. Ding, Prof. Dr. W. Wang
The Second Affiliated Hospital of Zhejiang University, Research Center of Diagnosis and Treatment Technology for Hepatocellular Carcinoma of Zhejiang Province, Hangzhou, Zhejiang 310009, China
B. Ye, Dr. Y. Ding, Prof. Dr. W. Wang
The Second Affiliated Hospital of Zhejiang University, Clinical Research Center of Hepatobiliary and Pancreatic Diseases of Zhejiang Province, Hangzhou, Zhejiang 310009, China
B. Ye, Dr. Y. Ding, Prof. Dr. W. Wang
Clinical Medicine Innovation Center of Precision Diagnosis and Treatment for Hepatobiliary and Pancreatic Disease, Zhejiang University, Hangzhou, Zhejiang 310009, China
B. Ye, Dr. Y. Ding, Prof. Dr. W. Wang
Cancer Center, Zhejiang University, Hangzhou, Zhejiang 310009, China
Prof. Dr. G. Yu
Key Laboratory of Bioorganic Phosphorus Chemistry & Chemical Biology, Department of Chemistry, Tsinghua University, Beijing 100084, P. R. China
H. Yang, Prof. Dr. Z. Mao
MOE Key Laboratory of Macromolecular Synthesis and Functionalization Department of Polymer Science and Engineering, Zhejiang University, Hangzhou, Zhejiang 310027, China
E-mail: zwmao@zju.edu.cn
Dr. H. Wu
Department of Gastroenterology, The Second Affiliated Hospital and Yuying Children's Hospital of Wenzhou Medical University, Wenzhou, Zhejiang, 325000, China
Prof. Dr. F. Huang
Stoddart Institute of Molecular Science, Department of Chemistry, Zhejiang University, Hangzhou, Zhejiang 310027, China
Zhejiang-Israel Joint Laboratory of Self-Assembling Functional Materials, ZJU-Hangzhou Global Scientific and Technological Innovation Center, Zhejiang University, Hangzhou, Zhejiang 311215, China

+ These authors contributed equally.

1. Materials and Methods

3-mercaptopropionic acid, trifluoroacetic acid (TFA), 1-cyclohexyl-2-(5H-imidazo[5,1-a]isoindol-5-yl)ethanol (NLG919), N-(3-dimethylaminopropyl)-N′-ethylcarbodiimide hydrochloride (EDC·HCl), 4-(dimethylamino)pyridine (DMAP), N-hydroxysuccinimide (NHS), 1,1′-carbonyldiimidazole, N-Boc-1,4-butanediamine, N-acetyl-D-penicillamine, acetic anhydride, triethylamine (TEA), tert-butylnitrite, (±)-S-nitroso-N-acetylpenicillamine (SNAP), glacial acetic acid, perchloric acid, kynurenine (Kyn) and p-dimethylamino-benzaldehyde were purchased from J&K (China) and Macklin (China). All reactants were purchased from commercial sources and used without further purification. Solvents were employed as purchased or dried over molecular sieves according to procedures described in the literature. Mono-6-deoxy-6-EDA-β-CD (β-CD-NH_2) and β-CD-Fc were synthesized according to the literature[1, 2]. CCK8 cytotoxicity assay kits, Griess reagent kit, LDH cytotoxicity assay kit, Annexin V-FITC apoptosis assay kit and ATP assay kit were bought from Beyotime Biotechnology Inc (Shanghai, P. R. China). Anti-CD11c-FITC, Anti-CD80-PE, Anti-CD86-APC, Anti-CD3-PerCP-Cy5.5, antiCD4-PE, anti-CD8-FITC, anti-CD45-APC-cy7, anti-IFN-γ-APC antibodies were all purchased from Becton, Dickinson and Company Inc. (New Jersey, USA). Antibodies for CRT, HMGB1, GSDME and caspase-3 were obtained from Abcam (UK). Tubulin was obtained from Cell Signaling Technology, Inc. (Boston, USA). IFN-γ ELISA Kit was purchased from Neobioscience Technology (Shenzhen, China). Millipore ultrapure water was obtained on a Milli-Q purification system.

NMR spectra were recorded on a Bruker ASCEND-400 MHz or Bruker Avance DMX-500 using a residual solvent signal as an internal reference. NMR-DOSY experiment was performed using bipolar longitudinal eddy current delay with gradients (LEDBPGP) sequence. Spectra were acquired with gradient pulses (δ) of 2 ms ranging in strength from 0.28 to 5.26 g/mm for the BBFO 5 mm NMR probe. A 50 to 200 ms diffusion delay was set and diffusion coefficient (*D*) was calculated from mono-exponential decays using Bruker Topspin 3.6 software. Mass spectrometry was obtained on the Agilent 6545 Quadrupole Time-of-Flight Mass Spectrometry (Q-TOF MS). Micro-syringe pump (model: LSP01-1A) was obtained from Longer Pump Company (China). DLS experiments were recorded on Malvern Zetasizer Nano-ZS with a 5 mW He-Ne laser at a wavelength of 633 nm to measure the particle size distribution of the objects in solution at room temperature. TEM investigations were carried out on a HT-7700

instrument. UV-vis spectra were performed with a HITACHI UH5300 UV/VIS spectrophotometer. Fluorescence images were observed using a CLSM (Leica TCS SP5) and flow cytometry analysis was analyzed by a BD FACS-CaliburTM flow cytometer. Absorbance was measured using a microplate reader (Tecan, Tecan Austria GmbH, Austria).

2. Nomenclature for Protons

Cyclodextrin carbohydrate unit:

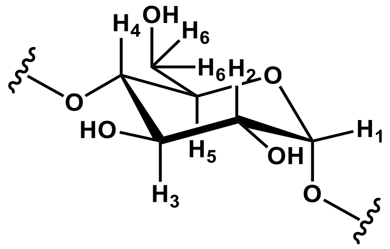

NLG moiety:

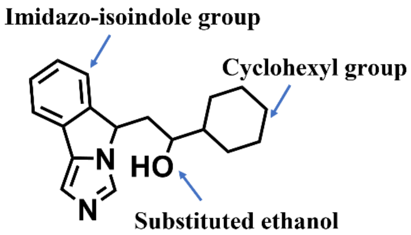

3. Experimental Section

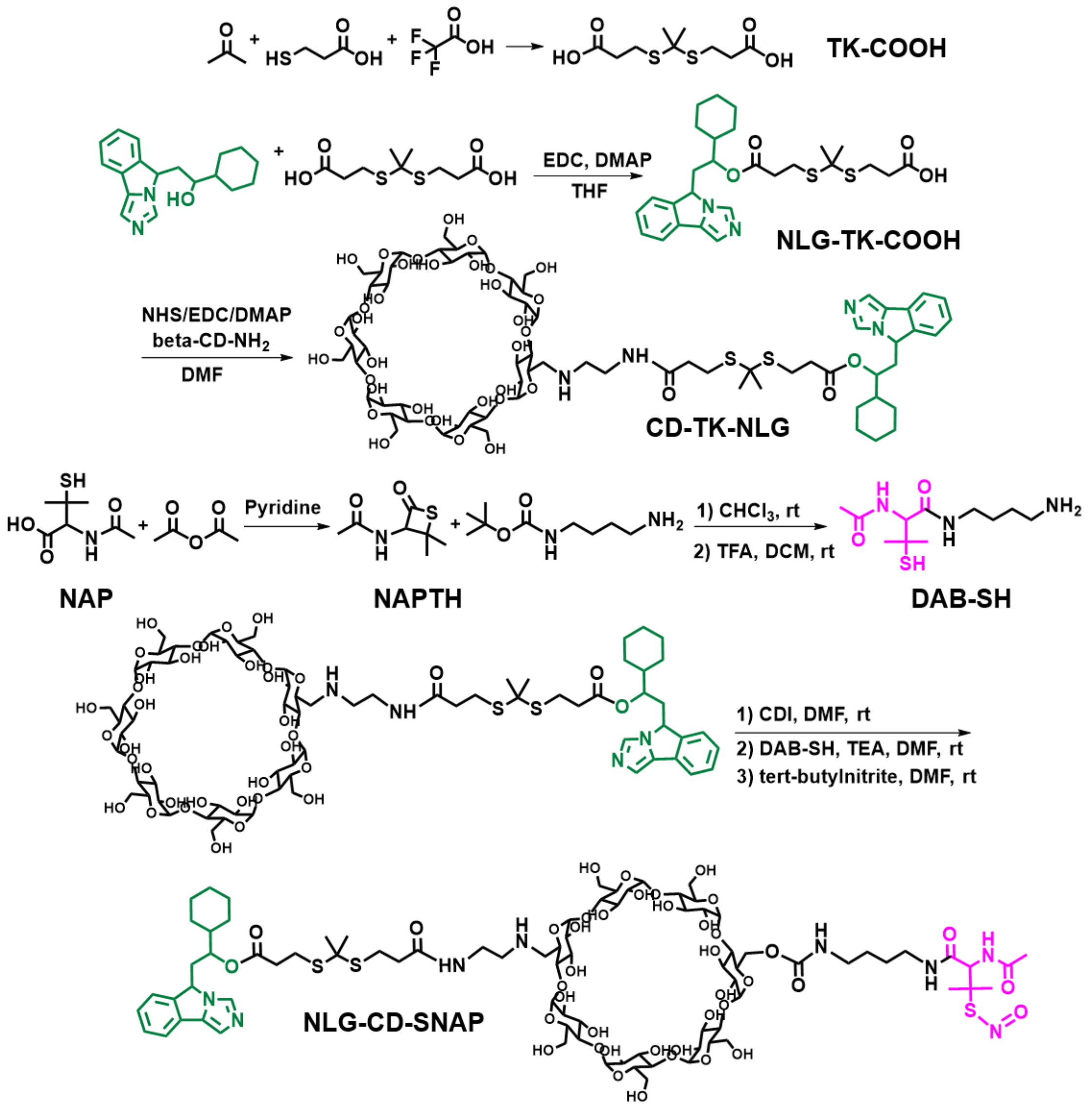

Scheme S1 Synthetic route to **NLG-CD-SNAP** (The number of donors was determined by NMR characterization).

Synthesis of TK-COOH. 3-mercaptopropionic acid (6.00 g, 56.8 mmol), acetone (1.50 g, 25.8 mmol) and a catalytic amount of TFA (0.29 g, 2.58 mmol) were added into a 20 mL vial under nitrogen atmosphere at room temperature for 6 h. After the reaction, the flask was sealed and chilled in ice until the crystallization completed. The crystals were filtered and washed with hexane and cold water to obtain TK-COOH as a white crystal (5.92 g, 90.7%) without further purification. **^{1}H NMR** (400 MHz, DMSO-d$_6$, 300 K): δ =12.29 (s, 2H), 2.73 (t, J=7.6 Hz, 7.8 Hz, 4H), 2.50 (t, J=7.2 Hz, 4H, overlaps with DMSO-d$_6$), 1.53 (s, 6H). MS (ESI) m/z calculated for $[C_9H_{16}O_4S_2\text{-}H]^-$ 251.0417; found 251.0410. The structure of the product was confirmed by comparison with the literature[3].

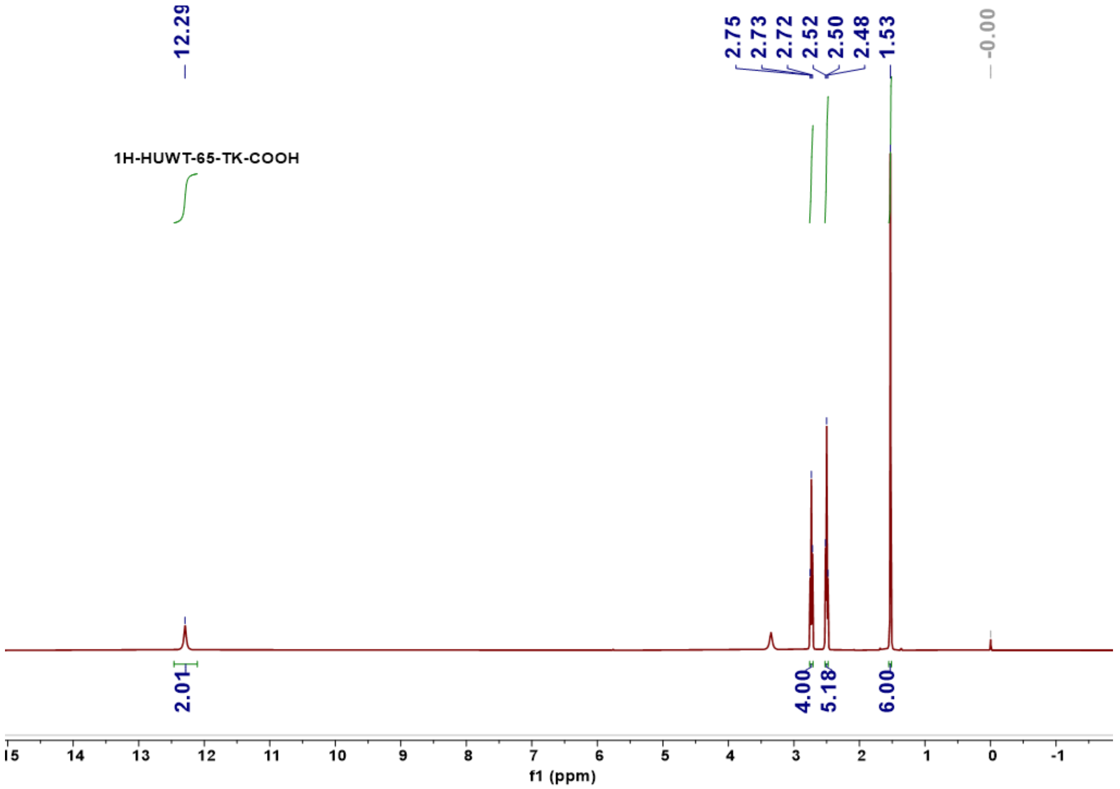

Figure S1 ^{1}H NMR spectrum (400 MHz, DMSO-d_6, room temperature) of TK-COOH.

Synthesis of NLG-TK-COOH. To a solution of NLG (564 mg, 2.0 mmol) in dry tetrahydrofuran (25 mL) was added TK-COOH (5.04 g, 20 mmol), EDC·HCl (383 mg, 2.0 mmol) and DMAP (48.8 mg, 0.4 mmol) under nitrogen and stirred at room temperature for 72 h. The reaction was concentrated under reduced pressure and poured into ammonium chloride solution (50 mL) and extracted by dichloromethane (100 mL). The organic layer was dried with anhydrous Na_2SO_4 and concentrated under reduced pressure. The residue was purified over silica gel chromatography (DCM/MeOH/CH_3COOH (200:1:0.1 then 100:1:0.1 then 50:1:0.1) to afford desired NLG-TK-COOH (324 mg, 31.5% yield) as the white solid. **^{1}H NMR** (400 MHz, CD_3OD, 300 K): δ =8.21 (s, 1H), 7.68-7.60 (m, 2H), 7.45-7.35 (m, 2H), 7.28 (s, 1H), 5.53-5.50 (m, 1H), 4.66 (ddd, J=10.1, 5.1, 2.4 Hz, 1H), 2.83-2.78 (m, 2H), 2.67-2.58 (m, 3H), 2.55 (t, J=7.2 Hz, 2H), 2.47 (ddd, J=15.1, 6.8, 2.4 Hz, 1H), 2.25 (dt, J=16.5, 7.2 Hz, 1H), 2.15-2.07 (m, 1H), 1.73 (s, 2H), 1.67-1.60 (m, 3H), 1.53 (d, J=1.7 Hz, 6H), 1.46-1.37 (m, 1H), 1.24-1.12 (m, 3H), 1.00-0.94 (m, 2H). **^{13}C NMR** (100 MHz, CD_3OD, 300 K): δ =174.81, 172.26, 145.15, 132.33, 129.83, 129.13, 127.82, 124.77, 120.98, 116.16, 73.58, 59.78, 56.22, 42.82, 35.84, 34.84, 34.35, 30.57, 29.18, 28.17, 26.73, 26.44, 25.74, 25.46. MS (ESI) m/z calculated for $[C_{27}H_{36}N_2O_4S_2+H]^+$ 517.2189; found 517.2188.

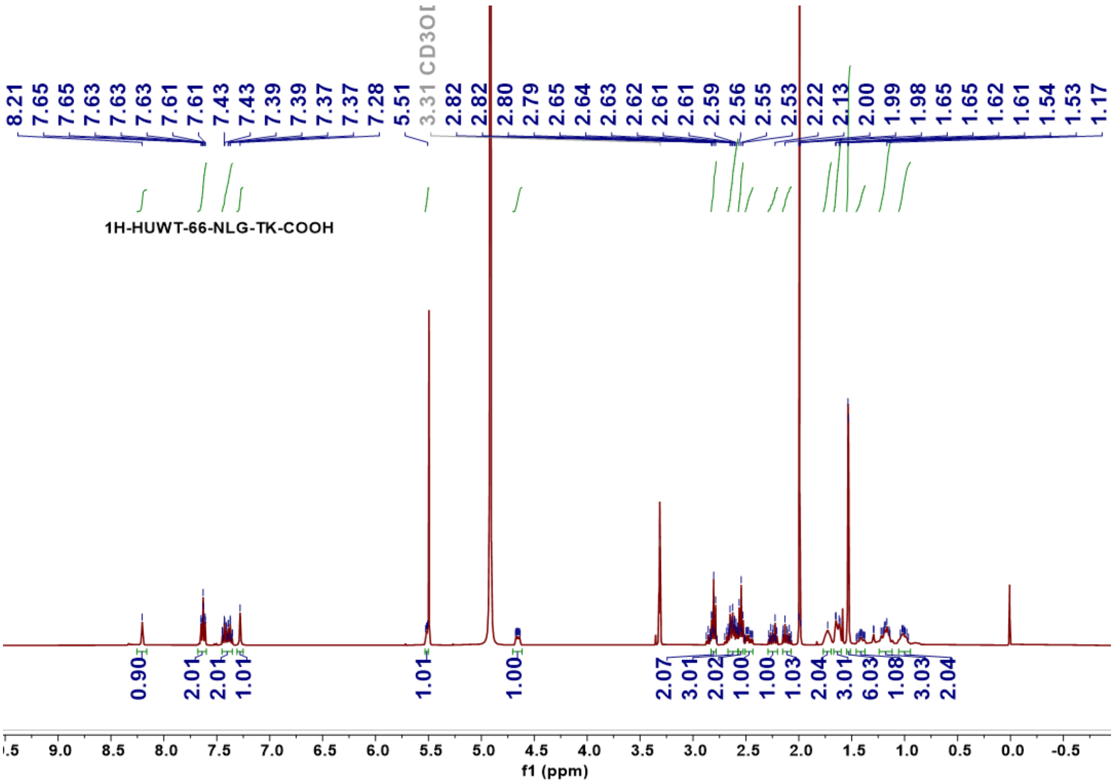

Figure S2 ^{1}H NMR spectrum (400 MHz, CD_3OD, room temperature) of NLG-TK-COOH.

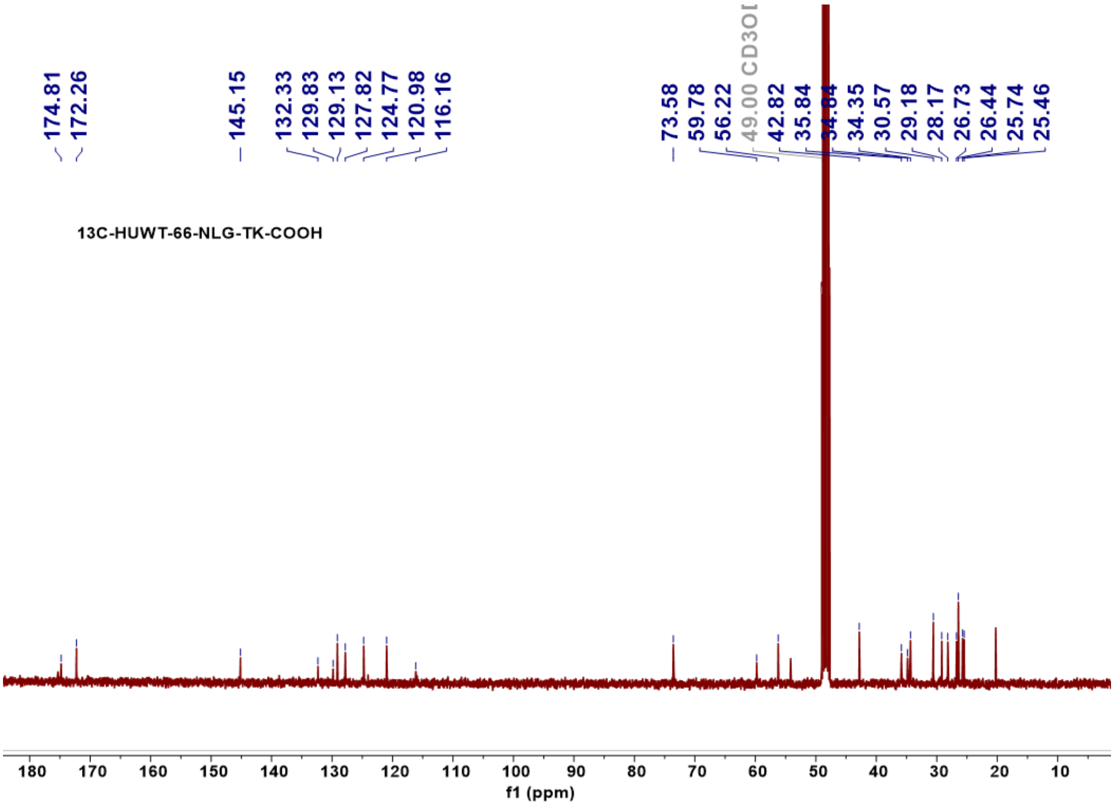

Figure S3 ^{13}C NMR spectrum (100 MHz, CD_3OD, room temperature) of NLG-TK-COOH.

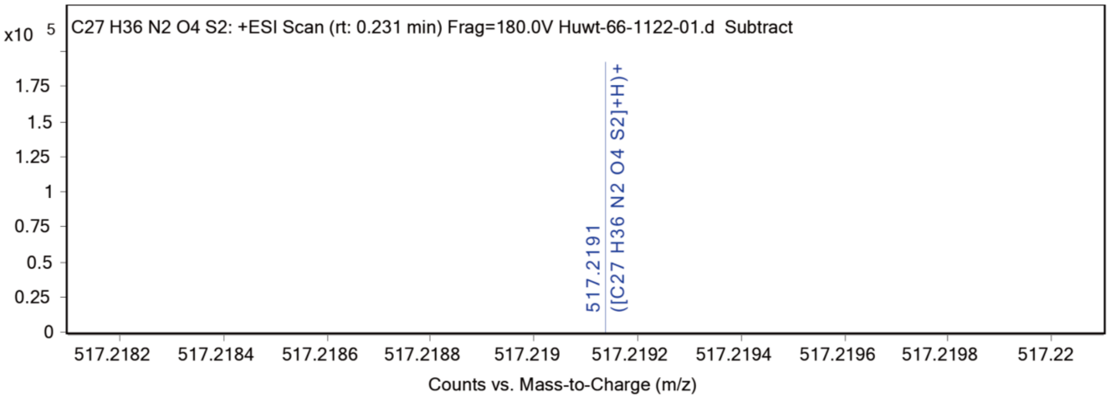

Formula Calculator Results

Formula	Best	Measured Mass	Tgt Mass	Diff (ppm)	Score
C27 H37 N2 O4 S2	True	517.2191	517.2189	-0.34	99.87

Figure S4 Electrospray ionization mass spectrum of NLG-TK-COOH.

Synthesis of CD-TK-NLG. A mixture of NLG-TK-COOH (250 mg, 1.0 eq), NHS (111 mg, 2.0 eq), EDC·HCl (278 mg, 3.0 eq) and DMAP (2 mg, 0.2 eq) in dry N, N-dimethylformamide (5 mL) was stirred under nitrogen. After being stirred for 24 h at room temperature, the reaction solution was poured into ethyl ether. The precipitate was collected by centrifugation, washed with cold ethyl ether several times, and dried under vacuum to give NLG-TK-NHS. β-CD-NH_2 (285 mg, 0.5 eq) was added to a solution of NLG-TK-NHS and TEA (30 μL) in dry N, N-dimethylformamide (5 mL). After being stirred for 24 h at room temperature, the solution was precipitated from chilled acetone, collected by centrifugation, dried under vacuum and finally collected by freeze-drying to give the final product of CD-TK-NLG (376 mg, 92.1%). **^{1}H NMR** (400 MHz, DMSO-d_6, 300 K): δ =10.04 (s, 1H), 7.68 (d, J=24.8 Hz, 2H), 7.44 (br.s, 1H), 6.11 (t, J=12.0 Hz, 1H), 6.02 (t, J=11.6 Hz, 1H), 5.99-5.51 (m, 9H), 5.04 (br.s, 1H), 4.93-4.68 (m, 5H), 4.66-4.34 (m, 4H), 3.97-3.45 (m, 19H), 3.43-3.19 (m, 26H, overlaps with water), 3.09-2.94 (m, 9H), 2.81-2.64 (m, 10H), 2.63-2.53 (m, 2H), 2.41-2.27 (m, 2H), 2.18 (d, J=8.4 Hz, 1H), 1.99-1.79 (m, 2H), 1.73 (dq, J=9.6, 6.8 Hz, 3H), 1.61 (d, J=34.2 Hz, 3H), 1.49 (s, 2H), 1.37 (br.s, 1H), 1.08 (m, 2H), 0.98 (t, J=7.2 Hz, 3H), 0.91 (d, J=12.3 Hz, 1H).

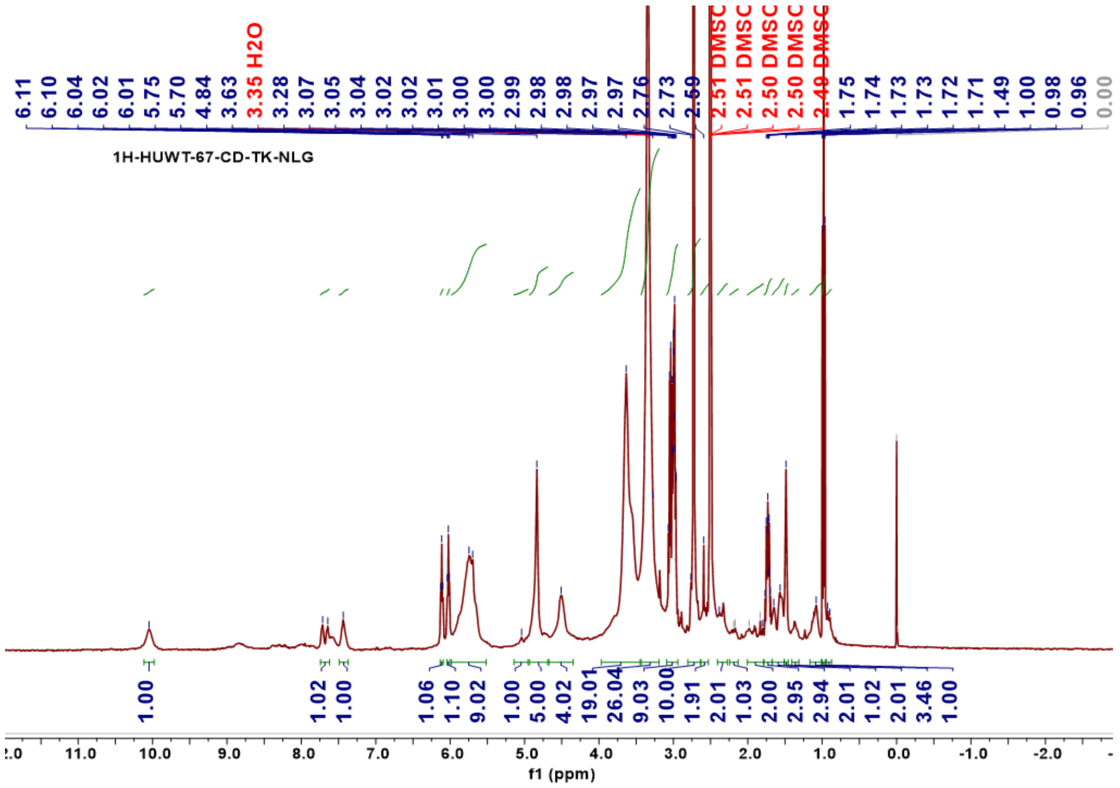

Figure S5 ^{1}H NMR spectrum (400 MHz, DMSO-d$_6$, room temperature) of CD-TK-NLG.

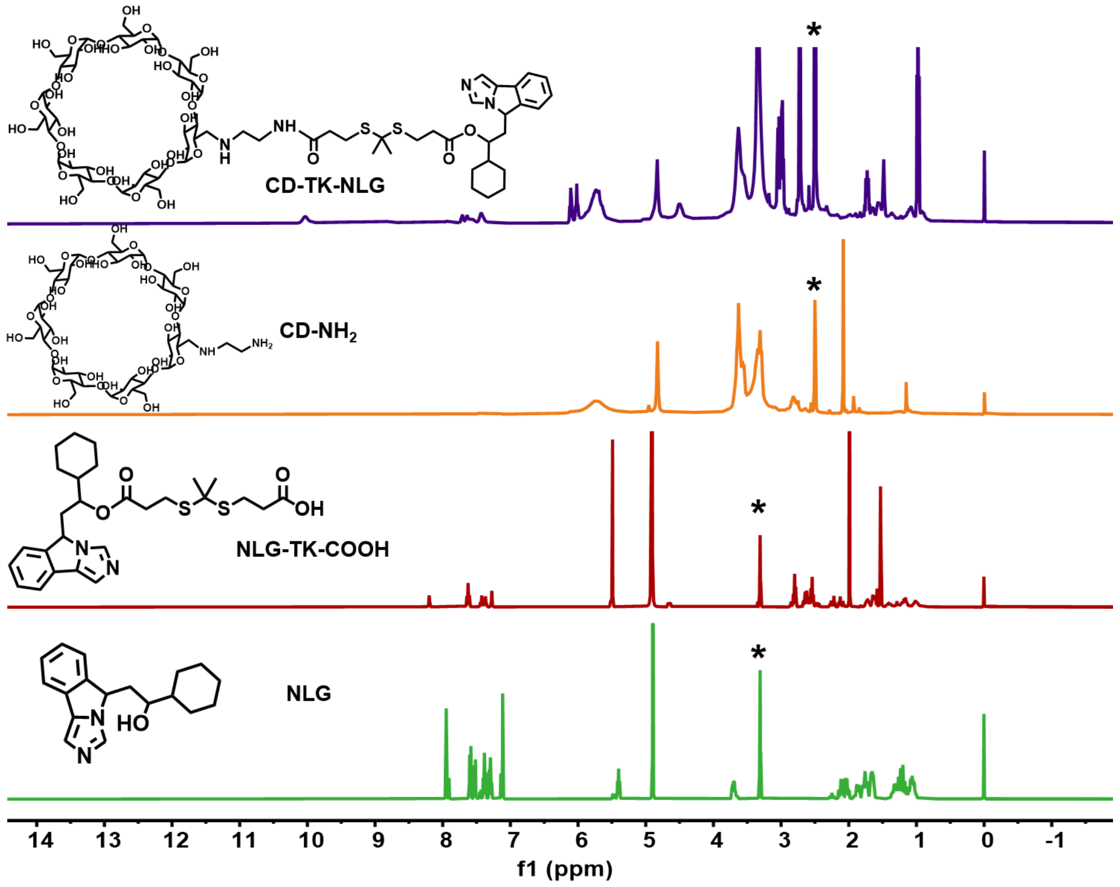

Figure S6 ^{1}H NMR spectra of CD-TK-NLG and CD-NH$_2$ in DMSO-d$_6$; NLG-TK-COOH and NLG in CD$_3$OD. The peaks of related solvents are marked with asterisks.

Synthesis of DAB-SH. To a cooled solution (0°C) of N-acetyl-D-penicillamine (NAP) (3.00 g, 15.7 mmol) in pyridine (10 mL) followed by acetic anhydride (3.97 mL, 42.1 mmol) in 20 mL of pyridine added dropwise. The mixture solution was stirred at 0°C under nitrogen atmosphere for 1 h and warmed to room temperature overnight. The redundant solvent was then removed by evaporation at reduced pressure until only an oily liquid remained. The raw product was dissolved in dichloromethane (10 mL) and washed with HCl solution (1 M in water, 3×10 mL). The organic layer was dried with Na_2SO_4, filtered and concentrated in vacuo. The obtained solid was dispersed in hexanes to participate and centrifuged to afford the N-acetyl-D-penicillamine thiolactone (NAPTH) as a white crystalline product (1.22 g, 44.6%). **^{1}H NMR** (400 MHz, $CDCl_3$, 300 K): δ =6.86 (s, 1H), 5.66 (d, J=7.2 Hz, 1H), 2.02 (s, 3H), 1.82 (s, 3H), 1.61 (s, 3H). **^{13}C NMR** (100 MHz, $CDCl_3$, 300 K): δ =192.67, 169.83, 76.39, 51.40, 30.32, 26.32, 22.66.

To a solution of NAPTH (600 mg, 3.46 mmol) in chloroform (25 mL) was added N-Boc-1,4-butanediamine (782 mg, 4.16 mmol) in one portion. After stirring at room temperature, the reaction solution was dried under reduced pressure. The residue was purified over silica gel chromatography (MeOH/DCM 1:30 then 1:10) to afford the desired Boc-DAB-SH (1.21 g, 92.0%) as the pale white solid. **^{1}H NMR** (500 MHz, $CDCl_3$, 300 K): δ =6.73 (s, 1H), 6.65 (d, J=9.5 Hz, 1H), 4.64 (s, 1H), 4.38 (d, J=9.0 Hz, 1H), 3.30-3.20 (m, 2H), 3.11 (br.m, 2H), 2.72 (s, 1H), 2.05 (s, 3H), 1.78 (br.m, 1H), 1.50 (br.m, 5H), 1.43 (br.m, 10H), 1.29 (s, 3H). **^{13}C NMR** (126 MHz, $CDCl_3$, 300 K): δ =170.73, 170.14, 156.49, 79.67, 60.69, 46.16, 40.39, 39.51, 31.53, 28.92, 28.81, 28.00, 23.70. MS (ESI) m/z calculated for $[C_{16}H_{31}N_3O_4S+Na]^+$ 384.1927; found 384.1930.

To a cooled solution (0 °C) of Boc-DAB-SH (5 g, 25.8 mmol, 1 eq) in anhydrous dichloromethane (10 mL) and followed by trifluoroacetic acid (965 uL, 12.6 mmol, 12 eq) added. The mixture solution was stirred at 0 °C under nitrogen atmosphere and allowed to warm to room temperature with stirring overnight. The solvent was then removed under reduced pressure. Cyclohexane was added and subsequently removed under reduced pressure 4 times. The residue was dissolved in dichloromethane and purified over silica gel chromatography through rapid washing with 10 mL aliquots of hexane, ethyl acetate, dichloromethane and a subsequent gradient of methanol in dichloromethane, to afford desired DAB-SH as the pale white oil. **^{1}H NMR** (500 MHz, D_2O, 300K): δ =4.27 (s, 1H), 3.23 (t, J=6.5 Hz, J=7.0 Hz, 2H), 2.97 (t, J=7.5 Hz, J=8.0 Hz, 2H), 2.05 (s, 3H), 1.65-1.62 (br.m, 2H), 1.59-1.55 (br.m, 2H), 1.43 (s, 3H), 1.39 (s, 3H). **^{13}C NMR** (126 MHz, D_2O, 300K): δ =174.13, 171.10, 62.79, 44.67, 39.02,

38.51, 29.57, 28.97, 25.34, 24.19. MS (ESI) m/z calculated for $[C_{11}H_{23}N_3O_2S+Na]^+$ 284.1403; found 284.1405.

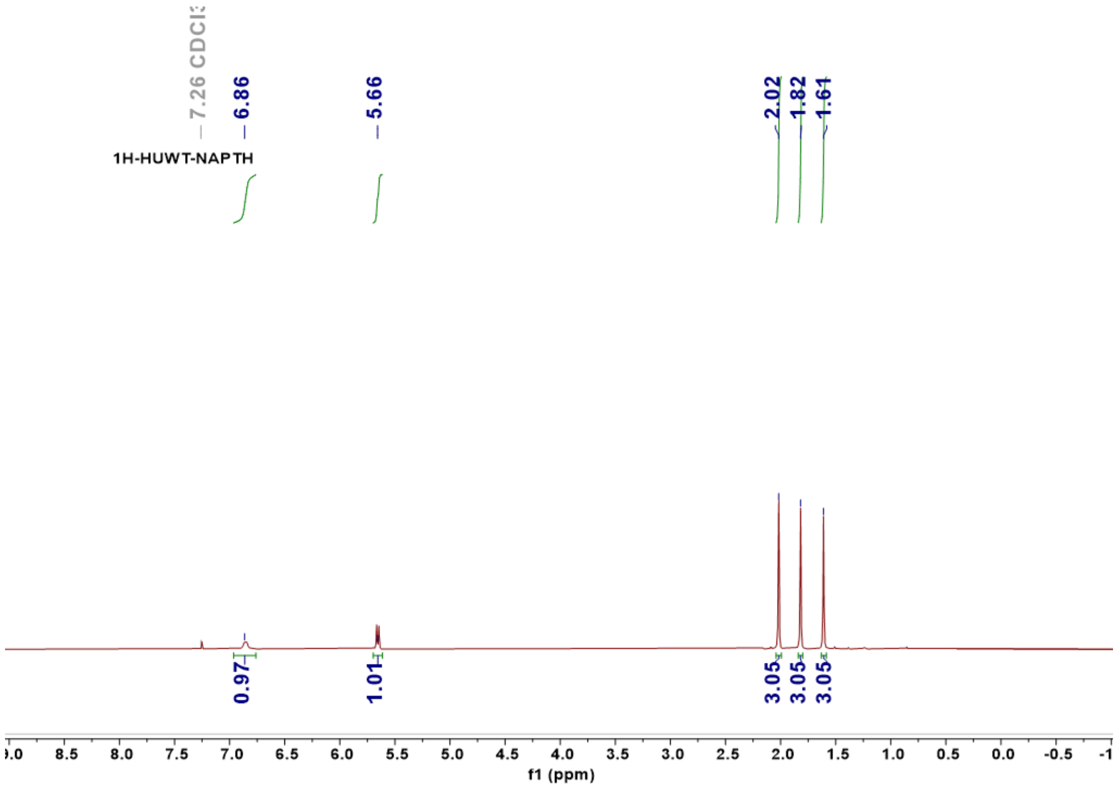

Figure S7 ^{1}H NMR spectrum (400 MHz, chloroform-d, room temperature) of NAPTH.

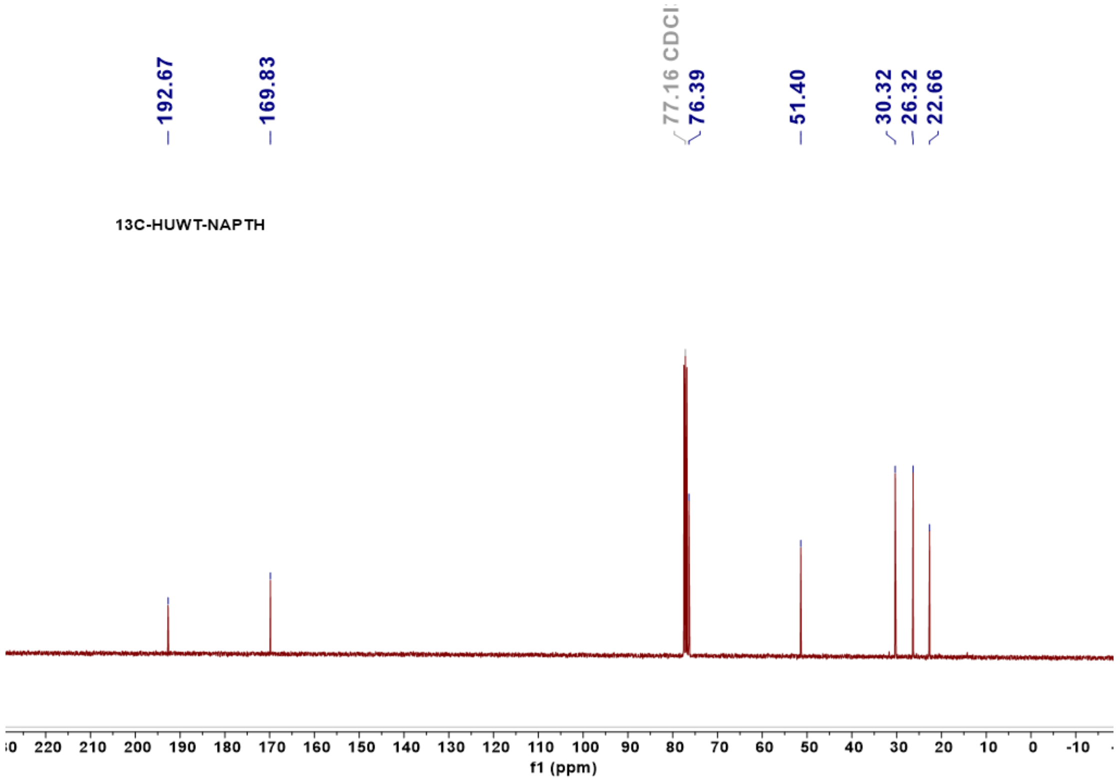

Figure S8 ^{13}C NMR spectrum (100 MHz, chloroform-d, room temperature) of NAPTH.

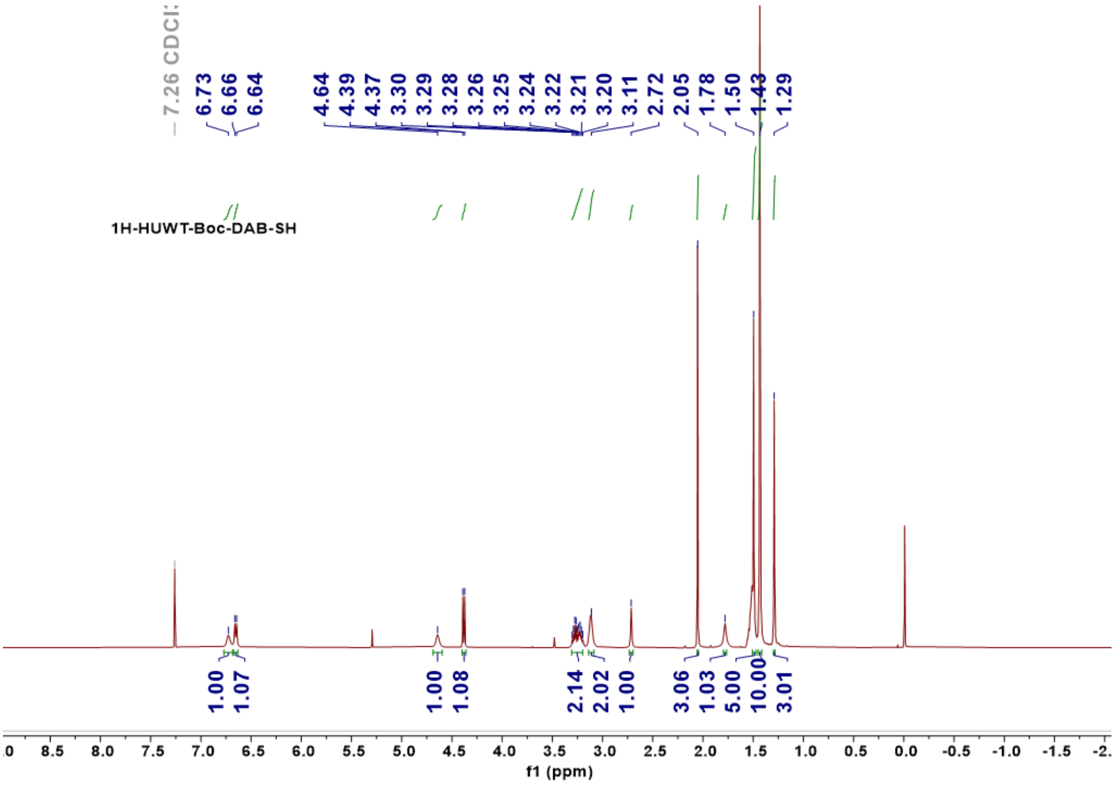

Figure S9 ^{1}H NMR spectrum (400 MHz, chloroform-d, room temperature) of Boc-DAB-SH.

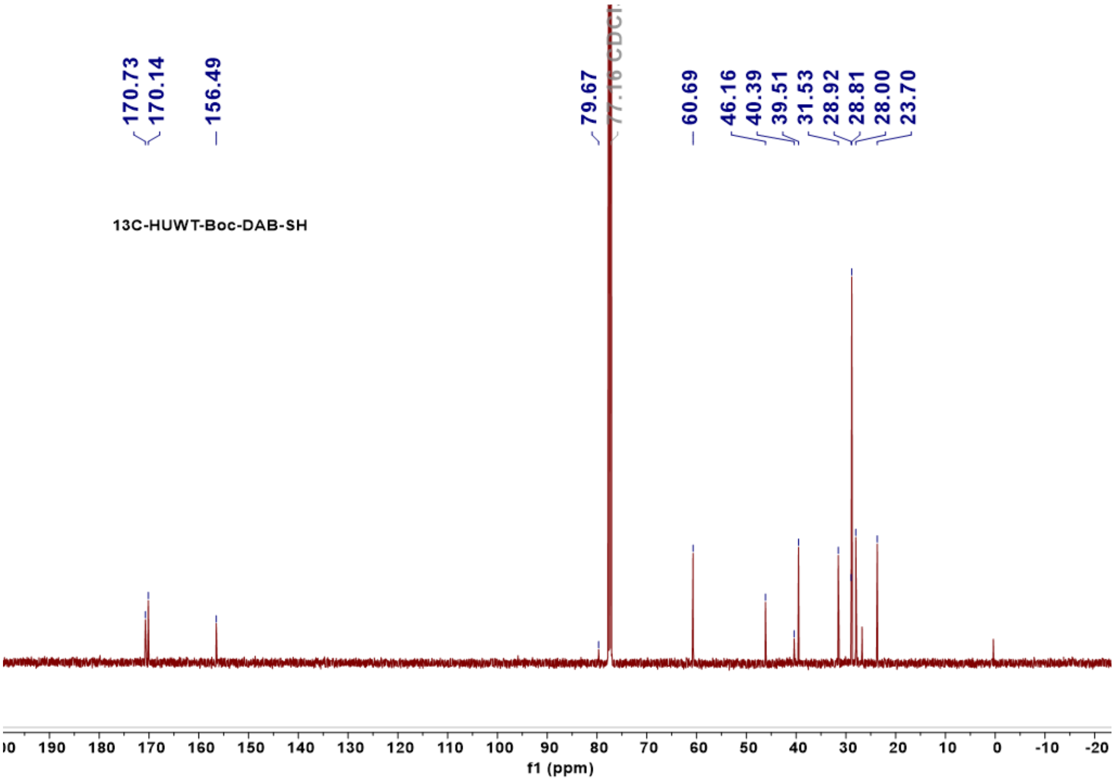

Figure S10 ^{13}C NMR spectrum (100 MHz, chloroform-d, room temperature) of Boc-DAB-SH.

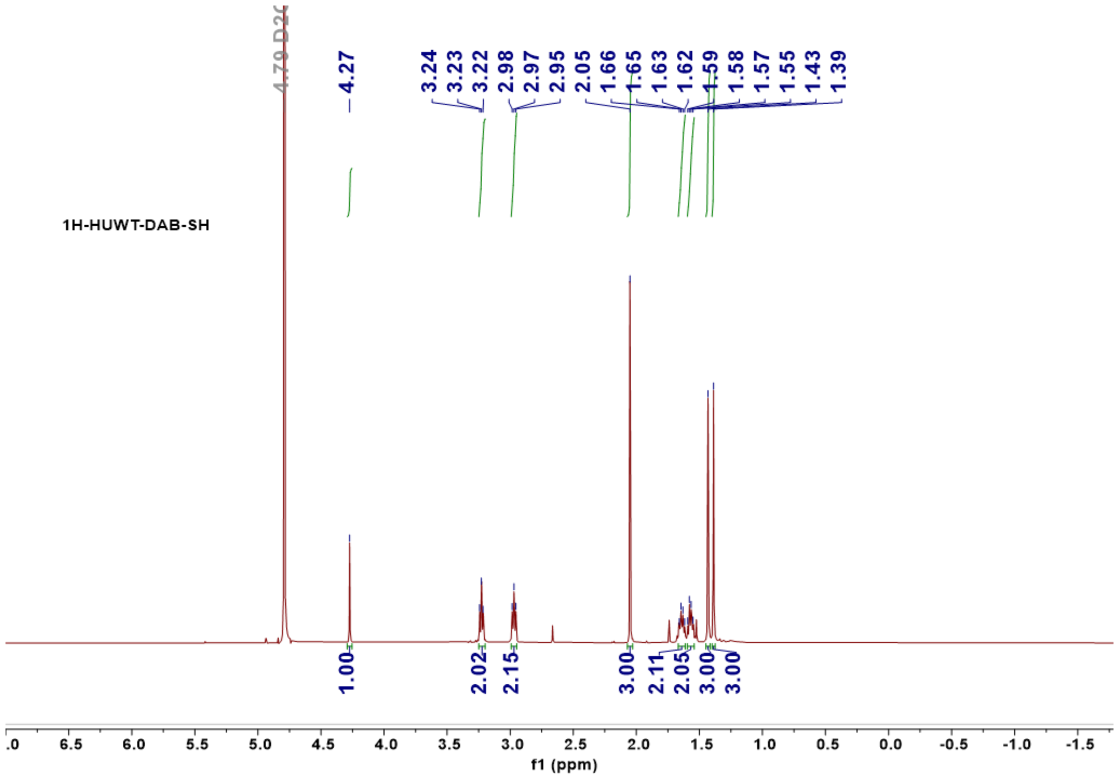

Figure S11 ^{1}H NMR spectrum (400 MHz, D_2O, room temperature) of DAB-SH.

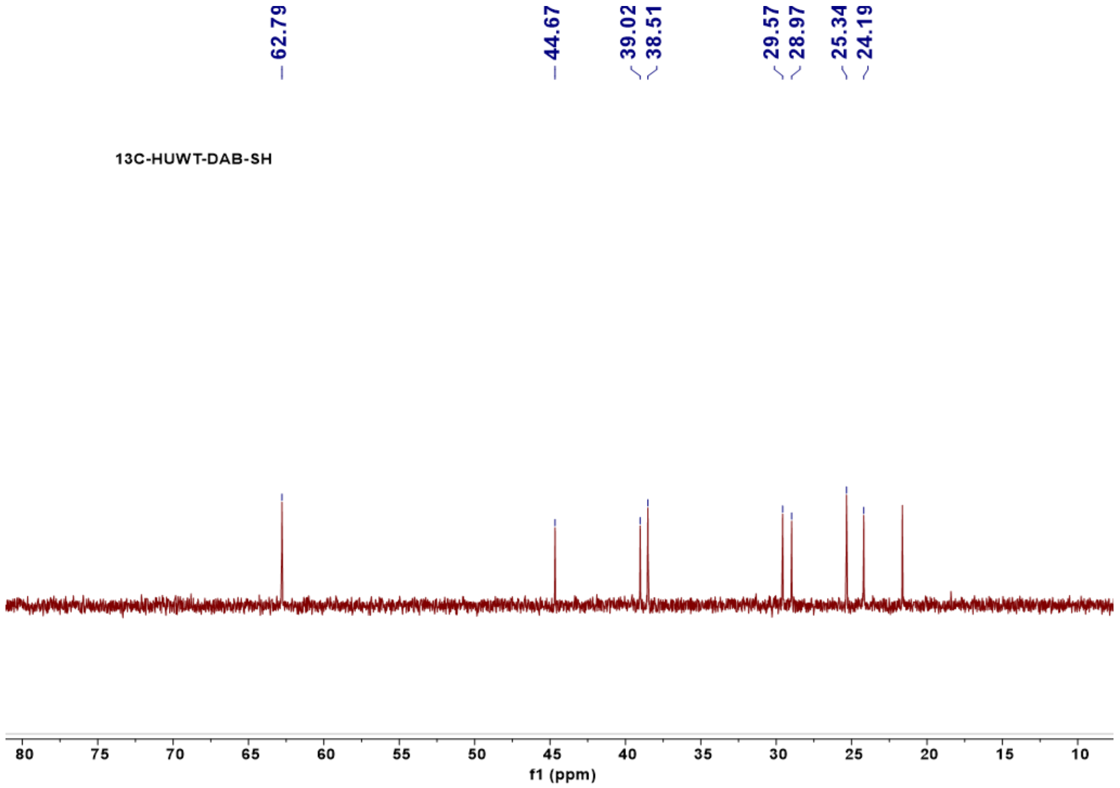

Figure S12 ^{13}C NMR spectrum (100 MHz, D_2O, room temperature) of DAB-SH.

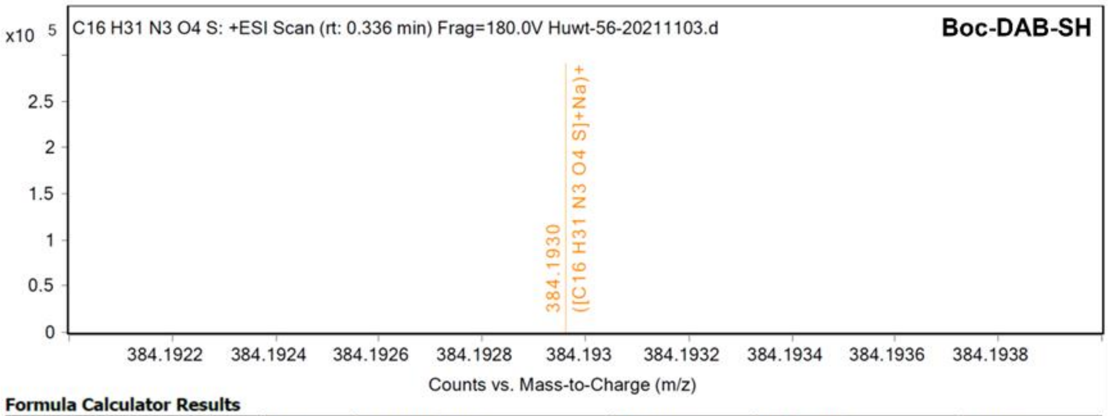

Formula Calculator Results

Formula	Best	Measured Mass	Tgt Mass	Diff (ppm)	Score
C16 H31 N3 Na O4 S	True	384.193	384.1927	-0.54	99.78

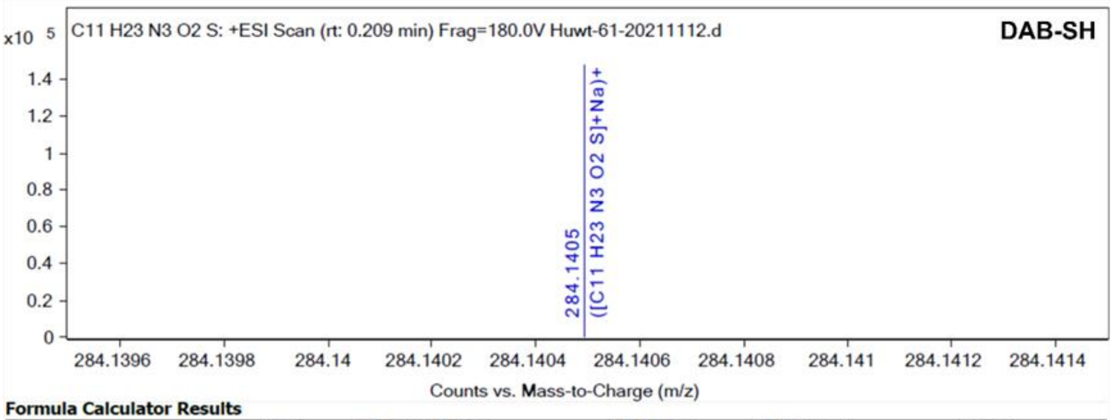

Formula Calculator Results

Formula	Best	Measured Mass	Tgt Mass	Diff (ppm)	Score
C11 H23 N3 Na O2 S	True	284.1405	284.1403	-0.67	99.76

Figure S13 Electrospray ionization mass spectra of Boc-DAB-SH and DAB-SH.

Synthesis of NLG-CD-SNAP. 1,1′-carbonyldiimidazole (CDI) (145 mg, 6 eq) and CD-TK-NLG (250 mg, 1 eq) were dissolved in 5 mL of dry N,N-dimethylformamide. The mixture was stirred at room temperature overnight under argon, and the solution was poured into chilled diethyl ether, collected by centrifugation, and dried under vacuum. Secondly, DAB-SH (117 mg, 3 eq) was added to a solution of obtained CDI-activated CD-TK-NLG and TEA (91 μL, 4.4 eq) in DMF (5 mL). After the mixture was reacted at room temperature for another 24 h, the product was precipitated from cold diethyl ether, and collected by centrifugation and freeze-drying. Finally, this product (100 mg, 50.9 μmol) and tert-butylnitrite (101 μL, 848 μmol) were dissolved in anhydrous N,N-dimethylformamide (5 mL) and stirred for 24 h at room temperature under argon atmosphere. The NLG-CD-SNAP was collected by being precipitated in cold diethyl ether, dried in vacuum, and obtained by freeze-drying as a pale-yellow fluffy powder.

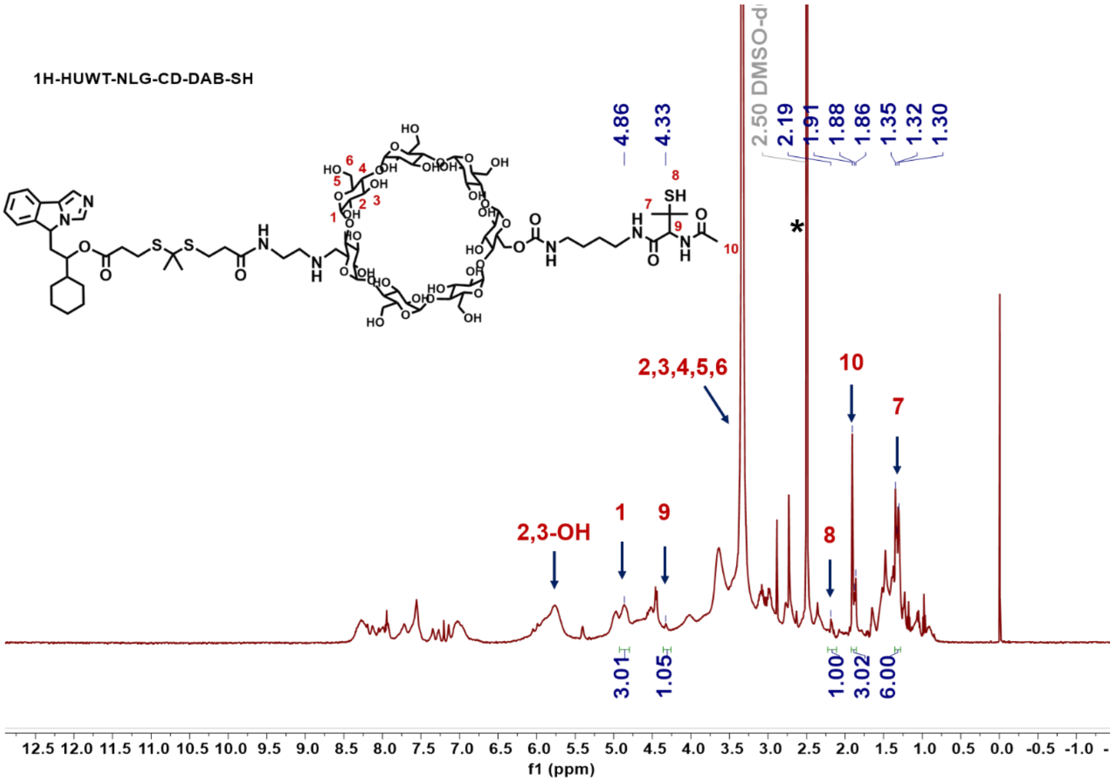

Figure S14 ^{1}H NMR spectrum (400 MHz, DMSO-d$_6$, room temperature) of NLG-CD-DAB-SH. The number of NO donors attached to the β-CD was determined by using the integral unit ratio of the ^{1}H NMR peak of the β-CD and the number was calculated to be 2.

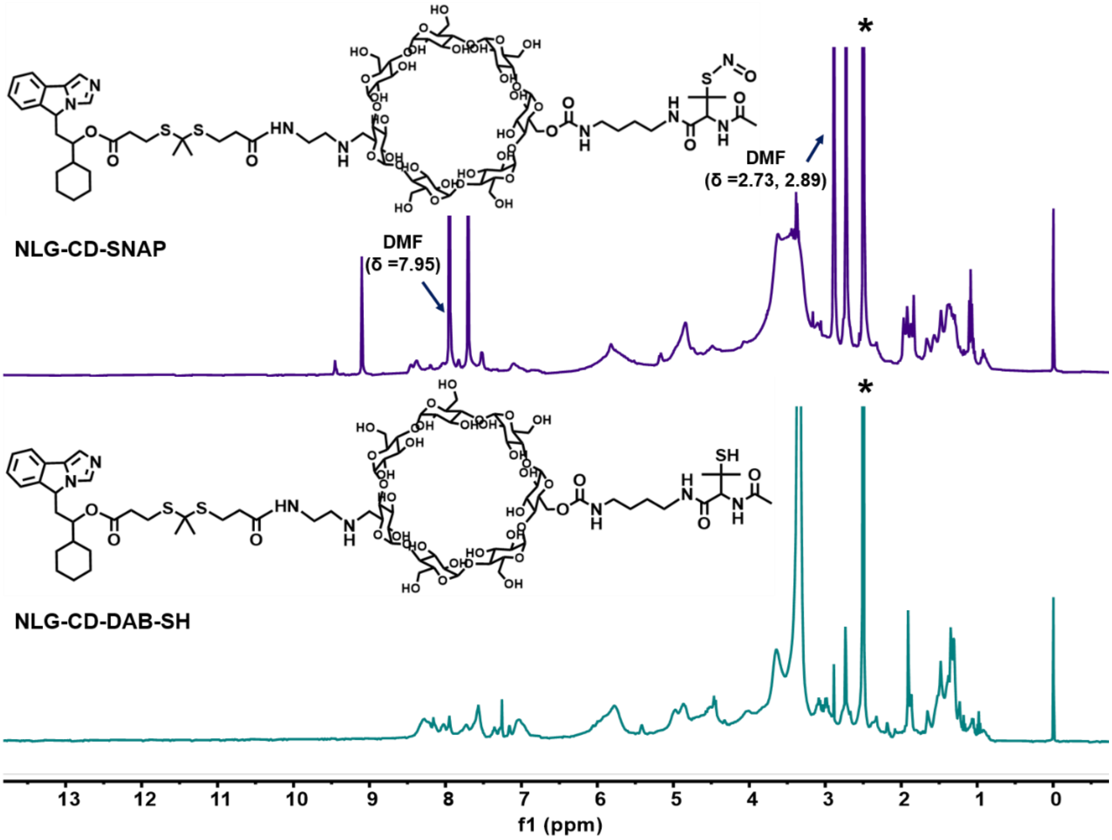

Figure S15 ^{1}H NMR spectra of NLG-CD-DAB-SH and NLG-CD-SNAP in DMSO-d$_6$. The peaks of related solvents are marked with asterisks.

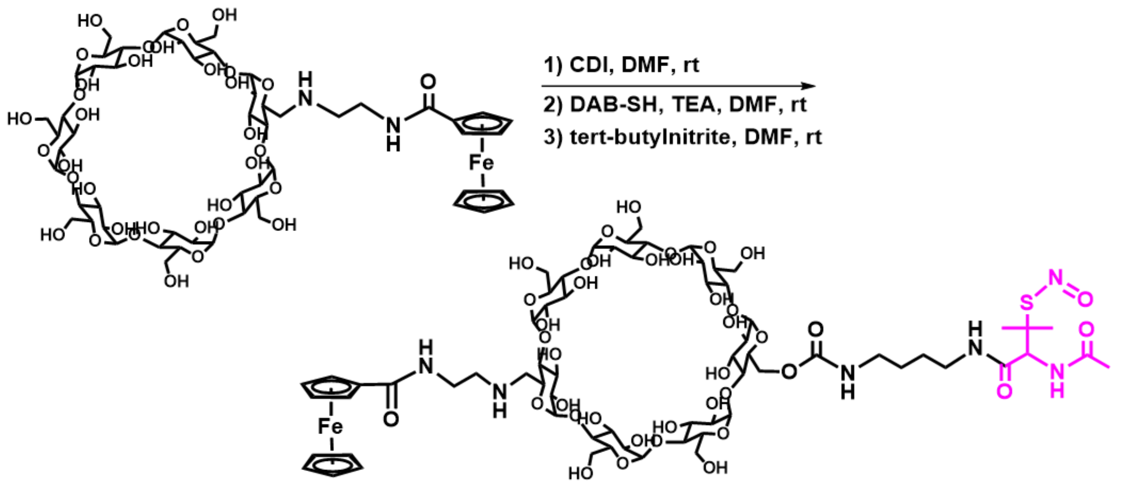

Scheme S2 Synthetic route to SNAP-CD-Fc (The number of NO donors was determined by NMR characterization).

Synthesis of SNAP-CD-Fc. 1,1′-carbonyldiimidazole (CDI) (140 mg, 6.0 eq) and CD-Fc (200 mg, 1.0 eq) were dissolved in 5 mL of dry N,N-dimethylformamide. The mixture was stirred at room temperature for 1.5 h under argon, and the product was precipitated from chilled diethyl ether and dried under vacuum. Then obtained CDI-activated β-CD-Fc was redissolved in 5 mL of dry N,N-dimethylformamide and then followed by DAB-SH (113 mg, 3.0 eq) and TEA (88 uL, 4.4 eq) sequentially added. After the mixture was reacted at room temperature for another 24 h, the reaction solution of Fc-CD-DAB-SH was precipitated from cold diethyl ether, collected by centrifugation, and finally obtained by freeze-drying. Fc-CD-DAB-SH (100 mg, 59.7 μmol) and tert-butylnitrite (118 uL, 993 μmol) were dissolved in anhydrous N,N-dimethylformamide (5 mL) and stirred for 24 h at room temperature under argon atmosphere. SNAP-CD-Fc was collected by being precipitated in cold diethyl ether and dried in vacuum.

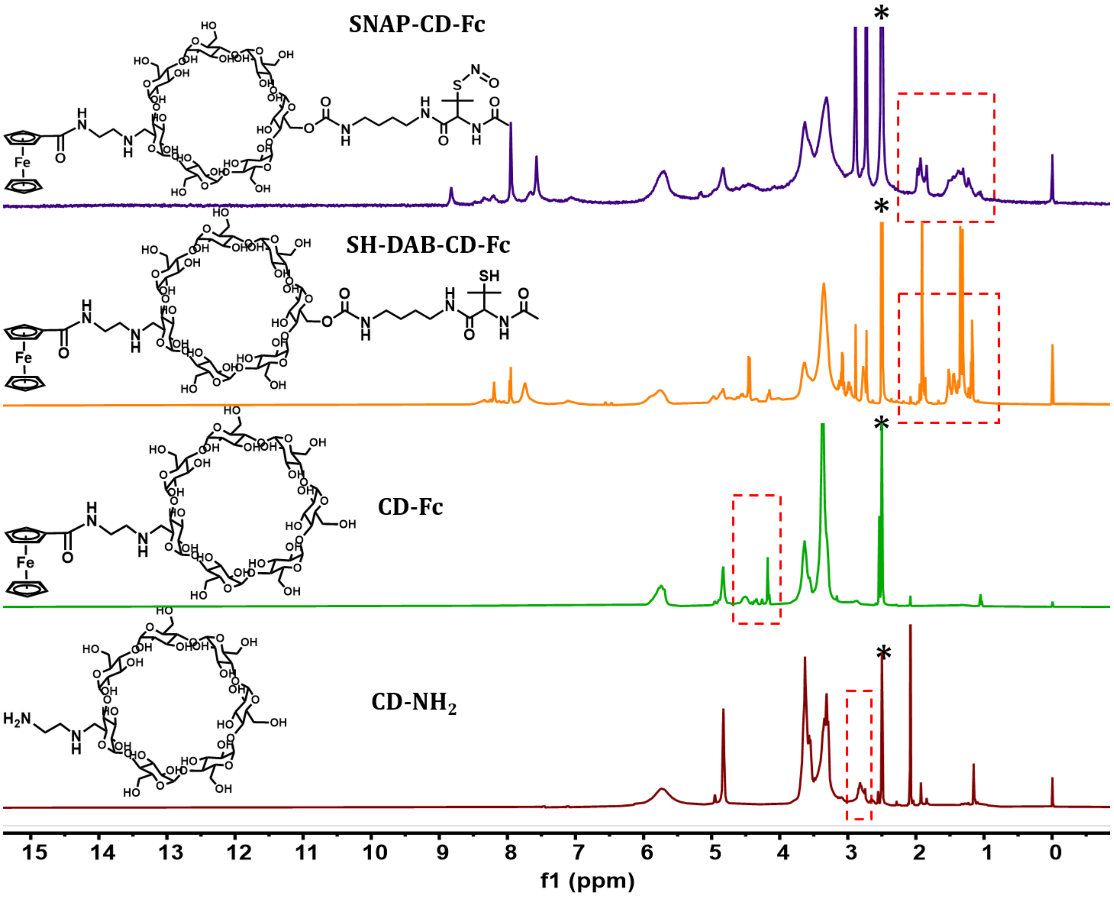

Figure S16 ^{1}H NMR spectra of CD-NH_2, CD-Fc, Fc-CD-DAB-SH and SNAP-CD-Fc in DMSO-d_6. The peaks of related solvents are marked with asterisks.

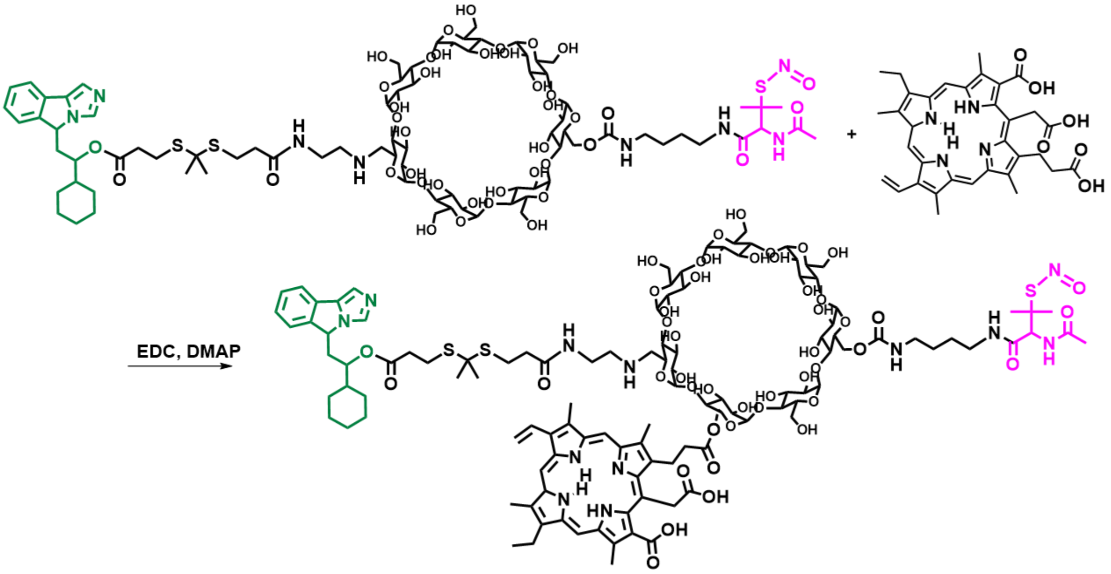

Scheme S3 Synthetic route to Ce6-NLG-CD-SNAP.

Synthesis of Ce6-NLG-CD-SNAP. The obtained NLG-CD-SNAP (20 mg, 1 eq) was dissolved in 5 mL of dry DMF and then followed by Ce6 (9.0 mg, 1.5 eq), EDC·HCl (7.7 mg, 4.0 eq) and DMAP (1.3 mg, 1 eq) sequentially added. After the mixture was reacted at room temperature for another 24 h, the reaction solution of Ce6-NLG-CD-SNAP was precipitated from cold

diethyl ether, collected by centrifugation and dried in vacuum, and finally obtained by freeze-drying.

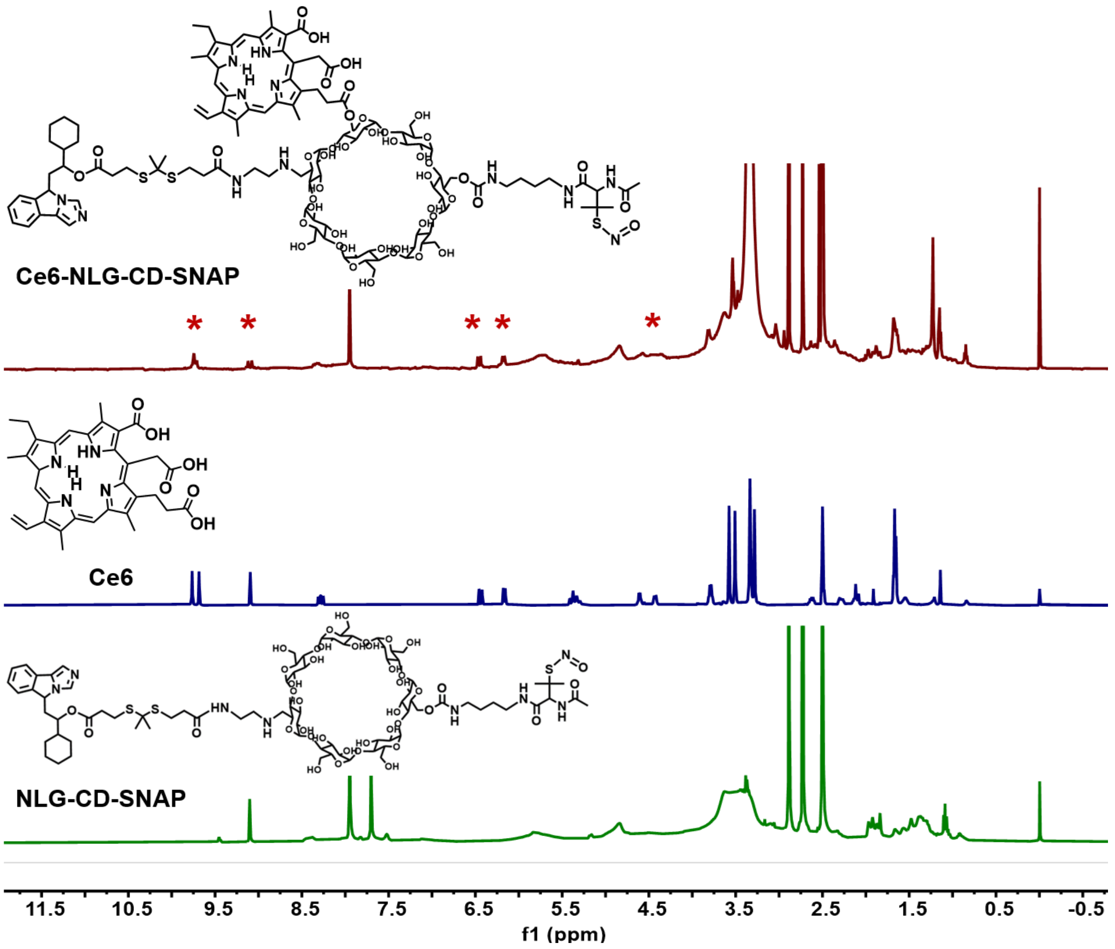

Figure S17 ^{1}H NMR spectra of Ce6-NLG-CD-SNAP, Ce6 and NLG-CD-SNAP in DMSO-d_6. The characteristic peaks of Ce6 from Ce6-NLG-CD-SNAP are marked with asterisks.

Fabrication of NCSNPs. The NCSNPs were prepared through the host-guest recognition between β-CD and NLG and hydrophobic interaction from the NO donor component. SNAP, an RSNO NO donor, could release NO through a reaction catalyzed by metal ions, heat or light. To avoid light and heat decomposition of SNAP, experiments were performed at 25 °C under dark conditions. Typically, NLG-CD-SNAP was dissolved in DMSO to prepare a stocking solution with different concentrations according to experimental requirements while stirring for 2h at 800 rpm in the dark. The deionized water was transferred to a disposable syringe fitted with a blunt-ended 23G stainless steel needle. The syringe was connected to a micro-infusion pump to maintain a constant flow rate of 0.1mL· min^{-1}. The resultant solution was stirred for another 12 h. The obtained solution containing NCSNPs was dialyzed against distilled water for 4~6 h and stored at 4°C for further use. IR780-labeled NCSNPs were prepared based on similar procedures. In this case, IR780 was co-dissolved with NLG-CD-SNAP in DMSO.

Stability evaluation of NCSNPs. The physiological stabilities of NCSNPs (1 mg·mL^{-1}) were carefully studied by recording their hydrodynamic diameters after being incubated in water, saline (0.9% NaCl) and FBS solution (10%) (2 mL) for varying time intervals.

Drug release studies of NCSNPs. The H_2O_2-sensitive drug release behaviors of NCSNPs were investigated using a dialysis strategy through monitoring the NLG release in various solutions. Briefly, the stocking solution was diluted with ultrapure water to a certain concentration solution (1 mg·mL^{-1}). The solution (1 mL) was transferred into a dialysis cassette (molecular weight cut-off=1000) and dialyzed against phosphate buffered saline (PBS) (10 mL) in the presence or absence of H_2O_2 (5 mM)[4], respectively. At scheduled intervals, 100 μL of the solutions were taken from dialysate for UV–vis spectrometer to measure the NLG concentration by recording the characteristic absorption peak of NLG at 272 nm. At the same time, fresh PBS with or without 5 mM H_2O_2 was put back into the dialysate. The NO release behaviors of NCSNPs in response to GSH were evaluated following the similar protocol described above. The NCSNPs solution was incubated with PBS in the presence or absence of GSH (10 mM)[5], respectively. And the NO release was measured by Griess assay. The controlled release experiments were calculated in triplicate.

Cell culture. B16 cells were cultured in RPMI 1640 with 10% FBS and 1% penicillin/streptomycin at 37°C in a 5% CO_2 incubator.

Confocal fluorescence imaging. The fluorescence microscope was used to determine the cellular uptake of NCSNPs. Firstly, B16 cells were seeded into 24-well plates at a density of 5×10^4 cells per well and incubated for 24 h. Subsequently, the cells were treated with Ce6-labeled NCSNPs for 0 h, 0.5 h, 1 h, 2 h, 4 h and 6 h, respectively. After rinsing the cells with PBS, the fluorescence images were further observed by confocal laser scanning microscopy.

Evaluation of cytotoxicity. The cytotoxicity of NCSNPs on B16 cells was evaluated by using the CCK8 assay. B16 cells were plated into 96-well plates at 8000 cells per well and cultured for 24 h for attachment. Then the culture medium was replaced with fresh media containing gradient concentrations of NLG, NCNPs, NCSNPs, NCSNPs + PTIO and incubated for another 24 h. After washing with fresh medium, the cells were incubated with a CCK8 solution (10 μL per well) for 2 h. The absorbance values of cells were measured at 450 nm using a microplate reader. Three replicate wells were tested per assay, and each experiment was performed in triplicate.

Intracellular NO detection. The intracellular NO detection was tested using a commercially

available DAF-FM solution (Beyotime S0019) as a photo-stable fluorescent probe to indicate NO. The experimental process follows: B16 cells were seeded into 24-well plates (2×10^4 cells per well) in DMEM medium and incubated for 24 h. The cells were treated with PBS, NCNPs, SCNPs, NLG plus SNAP and NCSNPs at an identical NLG concentration of 50 μM and SNAP concentration of 100 μM and incubated for 24 h, respectively. The cells were washed with PBS 3 times and stained with DAF-FM DA solution (1:1000 dilution) for another 30 min. Next, the medium was removed, and the cells were washed with PBS 3 times and fixed with 4% formaldehyde for another 20 min. The fluorescence images and quantitative data were obtained by CLSM and flow cytometry analysis.

***In vitro* pyroptosis assays**

Cell morphology assessment. The cells were treated with PBS, NCNPs, SCNPs, NLG plus SNAP and NCSNPs at an identical NLG concentration of 50 μM and SNAP concentration of 100 μM and incubated for 24 h to induce cell pyroptosis. The static bright-field images of pyroptotic cells were captured by optical microscopy. The cell swelling with large bubbles was observed to confirm the cell in the state of pyroptosis.

Annexin V-FITC/PI assay. Annexin V-FITC/PI assay was performed for B16 cells seeded into 6-well plates at a density of 1×10^5 and then pretreated with PBS, NCNPs, SCNPs, NLG plus SNAP and NCSNPs at an identical NLG concentration of 50 μM and SNAP concentration of 100 μM and incubated for 24 h. The cells were harvested to stain with Annexin V-FITC and PI according to the instruction manual. Fluorescence emission of Annexin V-FITC and PI was measured by flow cytometry. Annexin V-FITC green and PI red double-positive cells were considered to be pyroptotic cells.

LDH release assay. B16 cells were seeded into 96-well plates at a density of 1×10^5 and then pretreated with PBS, NCNPs, SCNPs, NLG plus SNAP and NCSNPs at an identical NLG concentration of 50 μM and SNAP concentration of 100 μM and incubated for 24 h. After centrifugation of 1000 rpm for 5 min, 120 μL of supernatant from each well was taken, then 60 μL LDH detection reagents were added to the supernatants and incubated for another 30 min at room temperature, and the optical density (OD) value was measured using a microplate reader. The relative LDH release was calculated by the ratio of OD value in the drug-treated group to that in the control group.

IDO inhibitory effect. To exploit the IDO inhibitory effect of NCSNPs, B16 cells were seeded in the 96-well plates at a density of 5000 cells per well. After 24 h incubation, IFN-γ was added

to each well to a final concentration of 100 ng·mL^{-1}. Meanwhile, the cells were treated with PBS, NCNPs, SCNPs, NLG plus SNAP and NCSNPs at an identical NLG concentration of 50 μM and SNAP concentration of 100 μM. After 24 h incubation, 150 μL of the supernatant from each well was incubated with perchloric acid (75 μL, 5%) to precipitate the proteins. Then the mixture was incubated with an equal volume of Ehrlich reagent (2% p-dimethylamino-benzaldehyde w/v in glacial acetic acid) at room temperature[6]. The absorbance of the reaction product was measured at 480 nm using a microplate reader.

Western blot assay. The levels of related proteins including tubulin, caspase-3, and GSDME in B16 cells from different groups were detected by Western blot assay. Briefly, B16 cells were pretreated with PBS, NCNPs, SCNPs, NLG plus SNAP and NCSNPs at an identical NLG concentration of 50 μM and SNAP concentration of 100 μM and incubated for 24 h. All the treated cells were washed and lysed in RIPA buffer containing phenylmethanesulfonyl fluoride. The extracted proteins were separated by SDS-PAGE and then transferred onto polyvinylidene difluoride (PVDF) membranes. The membranes were blocked in Protein Free Rapid Blocking Buffer for 30 min at room temperature and then incubated with indicated primary antibodies overnight at 4°C. After incubation with secondary antibodies, the membranes were imaged by a chemiluminescence system. Quantification of blots was performed by ImageJ software.

Immunogenic cell death induction

HMGB1 release assay. Intracellular HMGB1 distribution was performed on B16 cells seeded into 6-well plates (5×10^{6} cells per well) and tested by ELISA kits. After 24 h pre-incubation, the cells were incubated with PBS, NCNPs, SCNPs, NLG plus SNAP and NCSNPs at an identical NLG concentration of 50 μM and SNAP concentration of 100 μM and incubated for another 24 h. Next, the cell culture supernatant was collected and the HMGB1 content was tested using an ELISA kit according to the manufacturer's instructions. For immune-fluorescence analysis, cells were grown in 6-well glass-bottom plates at a density of 2 × 10^{4} per well. These cells were then treated with indicated drugs for a duration of 24 h. In order to test the intracellular distribution of HMGB1, the cells were fixed with 4% paraformaldehyde for 10 minutes. Following this, the cells were permeabilized with 0.1% Triton X-100 for 10 minutes. To prevent nonspecific binding, the cells were pre-incubated with 5% BSA in PBS for 30 minutes. After the pre-incubation, the cells were incubated with the primary antibody for a duration of 1 h. Subsequently, the cells were incubated with an Alexa Fluor 657-conjugated secondary antibody for a duration of 30 minutes after being washed three times with PBS. Finally, the cells were stained with Hochest33342 and examined using CLSM.

CRT express assay. The cell surface detection of CRT was performed by flow cytometric measurement. B16 cells were seeded in the 12-well plates at a density of 1×10^5 cells per well for 24 h. The cells were treated with PBS, NCNPs, SCNPs, NLG plus SNAP and NCSNPs at an identical NLG concentration of 50 μM and SNAP concentration of 100 μM and incubated for another 24 h. Subsequently, the cells were washed twice with cold PBS and fixed in 0.25% paraformaldehyde for 5 min. After washing twice in cold PBS, the cells were incubated with primary antibody. The cells were rewashed 30 min later and incubated with the Alexa488-conjugated monoclonal secondary antibody for 30 min. Finally, the cells were analyzed by flow cytometry.

ATP release assay. Extracellular secretion of ATP was tested using a commercially available ATP assay kit. Briefly, B16 cells were seeded in the 24-well plate at a density of 4×10^4 cells per well. After 24 h pre-incubation, the cells were incubated with PBS, NCNPs, SCNPs, NLG plus SNAP and NCSNPs at an identical NLG concentration of 50 μM and SNAP concentration of 100 μM and incubated for another 24 h. Then, the cell culture supernatant was collected and the ATP content was tested using an ATP assay kit according to the manufacturer's instructions.

DC maturation *in vitro*. To investigate DC maturation *in vitro*, BMDCs were generated from the bone marrow of 8-week-old C57BL/6 mice. B16 cells were pre-treated with PBS, NCNPs, SCNPs, NLG plus SNAP and NCSNPs at an identical NLG concentration of 50 μM and SNAP concentration of 100 μM and incubated for 24 h. Subsequently, 1×10^6 immature DC cells were co-cultured with 1×10^5 pre-treated B16 cells. After staining with Live/Dead FSV510, anti-CD11c-FITC, anti-CD80-PE and anti-CD86-APC antibodies, the maturation of DC cells was examined using flow cytometry measurement.

Animals and tumor models. Five-week-old female C57BL/6 mice were purchased from the Zhejiang Academy of Medical Sciences and maintained in a pathogen-free environment under a controlled temperature (24°C). All animal experiments were carried out by the Institute of Laboratory Animal Resources guidelines. Ethical approval was granted by the Institutional Animal Care and Use Committee of the Second Affiliated Hospital, School of Medicine, Zhejiang University, China. The mice were injected subcutaneously in the right back region with 50 μL of cell suspension containing 1×10^7 B16 cells on day −7. The primary tumors were allowed to grow to ~100 mm^3 before experimentation. The tumor volume was calculated as (tumor length) × (tumor width)^2/2.

***In vivo* combination therapy.** The C57BL/6 mice bearing B16 tumors were randomly divided

into five groups and intravenously injected with saline, NCNPs (20.0 mg NLG/kg), SCNPs (31.2 mg SNAP/kg), NLG (20.0 mg/kg) plus SNAP (31.2 mg/kg) and NCSNPs (20.0 mg NLG/kg; 31.2 mg SNAP/kg) every 2 days for three times, respectively. Tumor volume and body weight were measured every 2 days. The tumors were harvested at the end of antitumor studies. The blood was collected for biochemical assay. In the histological assay, the main organs (including the heart, liver, spleen, lung, and kidney) were fixed in 4% paraformaldehyde for 24 h. The specimens were dehydrated in graded ethanol, embedded in paraffin, and cut into 5 mm thick sections. The fixed sections were deparaffinized and hydrated according to a standard protocol and stained with hematoxylin and eosin (H&E) for microscopic observation. Pyroptosis of the mice's tumor cells after treatments were determined by the TUNEL method according to the manufacturer's instructions. Detection of the tumor cell proliferation in the mice after treatments were determined by the Ki67 method according to the manufacturer's instructions.

Intratumoral infiltration of T lymphocytes. To examine the intratumoral infiltration of T lymphocytes, the tumors were harvested on day 6 and cut into small pieces and immersed in the solution of 1 mg·mL^{-1} collagenase IV and 0.2 mg·mL^{-1} DNase I for 45 min at 37°C, and the small tumor pieces were pressed gently to obtain a single cell suspension solution. Finally, the single cells were stained with a fluorescent-labeled antibody according to the manufacturer's protocols. For the analysis of CTLs ($CD3^+CD4^-CD8^+$) and $CD4^+$ T cells ($CD3^+CD4^+CD8^-$), the lymphocytes were stained with Fixable Viability Dye (FSV510)-AmCyan-A, anti-CD45-APC-Cy7, anti-CD3-PerCP-Cy5.5, anti-CD4-FITC, anti-CD8-PE antibodies according to the manufacturer's protocols. The cells were analyzed by flow cytometric measurement.

Kyn and IFN-γ measurement. To measure Kyn in the tumor site, the tumors were harvested on day 6 and cut into small pieces and homogenized in protein lysate buffer. The tumor tissue homogenate was dissolved with an equal volume of perchloric acid (5%) to precipitate the proteins. After centrifugation, the collected supernatant (50 μL) was mixed with 50 μL Ehrlich reagent (2% p-dimethylamino-benzaldehyde w/v in glacial acetic acid), and incubated for 10 min at room temperature. Absorbance at 480 nm was determined on a microplate reader. Meanwhile, a series of Kyn dissolved in water containing 2.5% perchloric acid with different concentrations was used to draw a standard curve. IFN-γ secretion in sera was measured using an ELISA kit according to the manufacturer's instructions.

***In vivo* imaging and bio-distribution.** To analyze the bio-distribution of the NCSNPs *in vivo*, the mice were randomly divided into five groups (three per group) and intravenously injected

with IR780-labeled NCSNPs (NCSNPs 2 mg·mL^{-1} and IR780 100 μg·mL^{-1}, 200 μL) at the predetermined times. Then, the mice were sacrificed to collect their tumors for distribution analysis by IVIS Lumina II *in vivo* imaging system at 1, 2, 4, 8, and 24 h post-injection. The fluorescence intensity was evaluated by ROI tools.

Blood biochemical assay. The blood sample of each mouse was collected from the retro-orbital plexus into a coagulation-promoting tube, and then centrifuged at 5000 rpm for 10 min to obtain serum samples for measuring clinic parameters including alanine aminotransferase (ALT), serum albumin (ALB), creatinine (CREAT) and blood urea nitrogen (BUN).

4. Statistical Analysis

GraphPad Prism 8.0 (GraphPad Software, CA, USA) was utilized to perform statistical comparisons. One-way ANOVA followed by Tukey's post-hoc multiple comparison test and unpaired t-test were used to analyze the significance of differences in IHC staining. The results are presented as the mean ± standard error of the mean and statistical significance was defined as $P < 0.05$ (* $P < 0.05$, ** $P < 0.01$, *** $P < 0.001$).

5. Supporting Figures

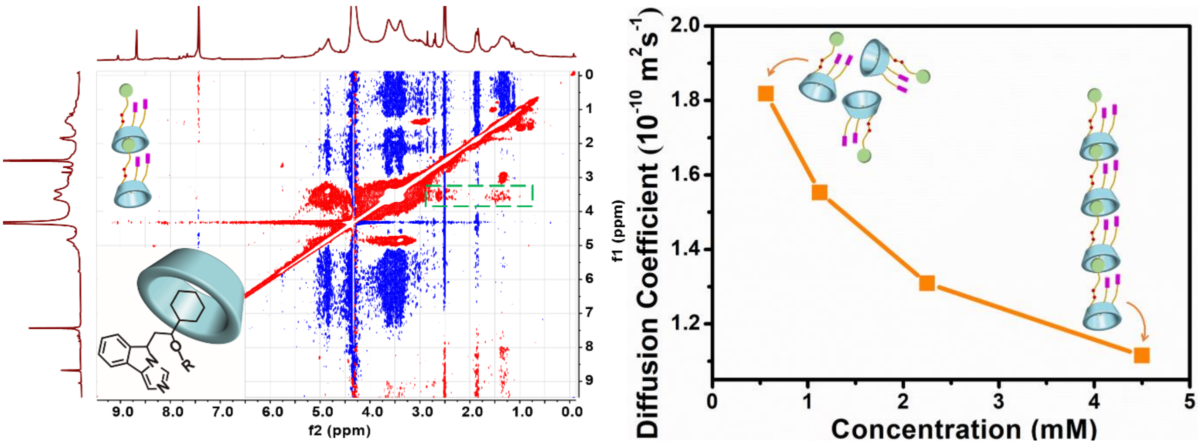

Figure S18 2D NOESY spectrum (400 MHz, room temperature) of NLG-CD-SNAP (left). Concentration dependence of the diffusion coefficient for NLG-CD-SNAP (right) (from ^{1}H NMR spectroscopy, 500 MHz, D_2O, 300 K).

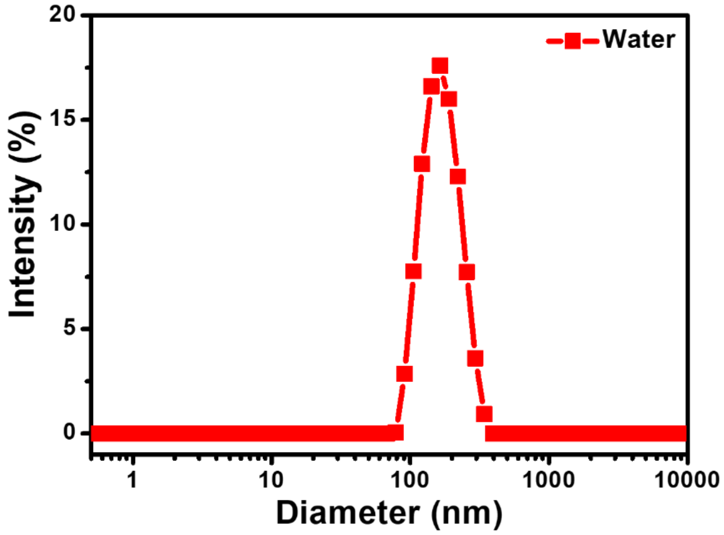

Figure S19 Hydrodynamic diameters of NCSNPs by DLS.

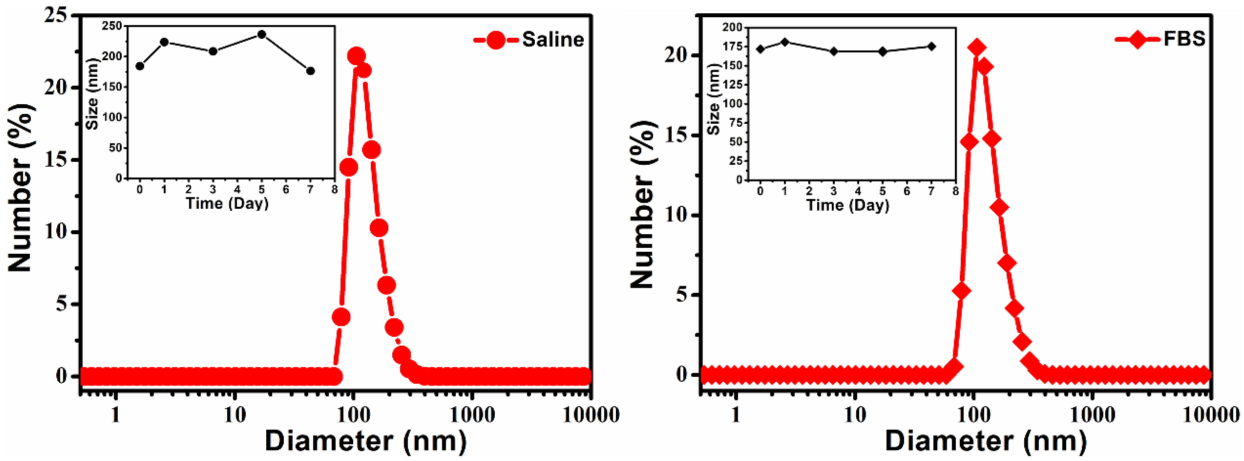

Figure S20 Hydrodynamic diameters of NCSNPs incubated in saline and FBS.

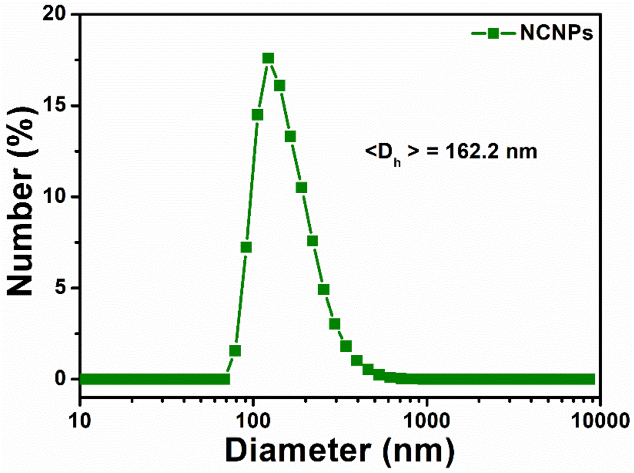

Figure S21 Hydrodynamic diameter of NCNPs by DLS.

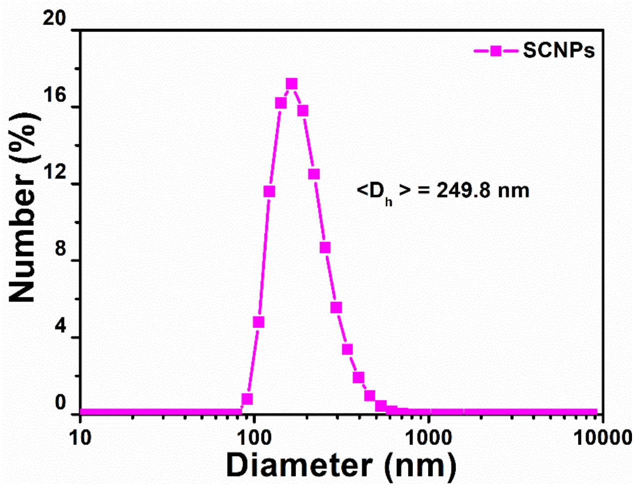

Figure S22 Hydrodynamic diameter of SCNPs by DLS.

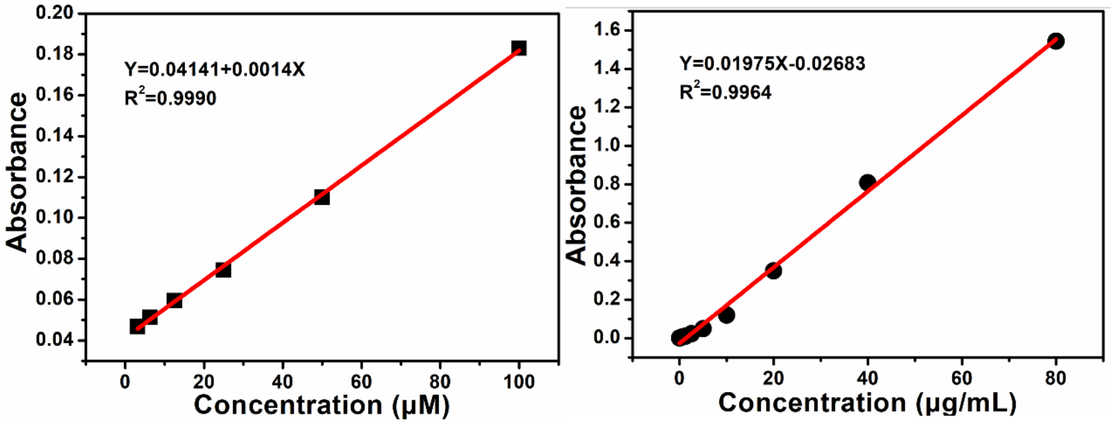

Figure S23 The standard curve of NO (left) and NLG (right) involved in drug release experiments.

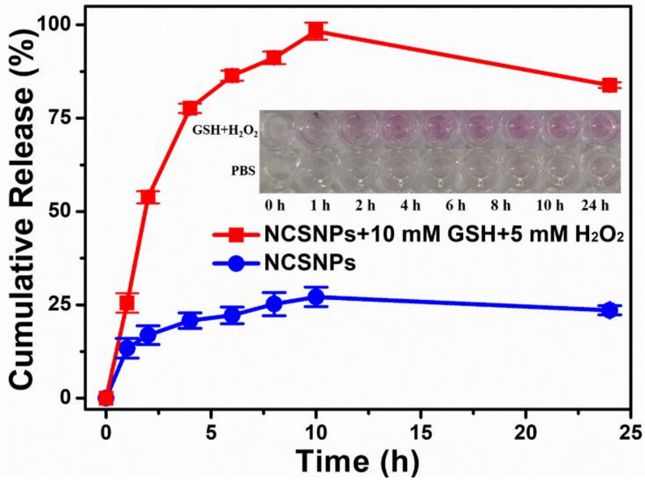

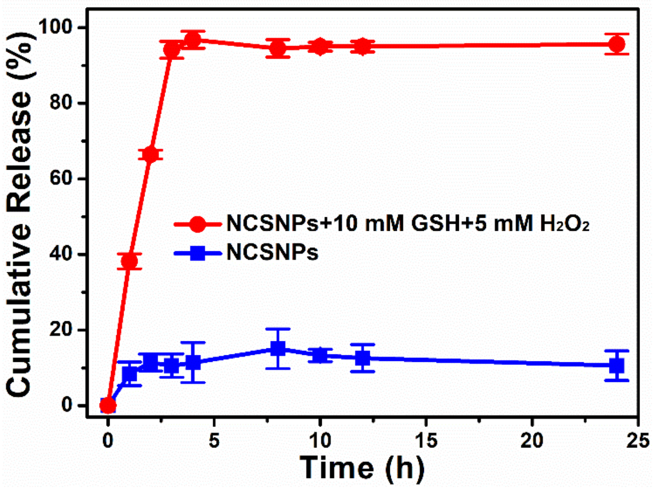

Figure S24 Release of NO donor (up) and release of NLG prodrug (down) from NCSNPs in the presence and absence of 10 mM GSH and 5 mM H_2O_2.

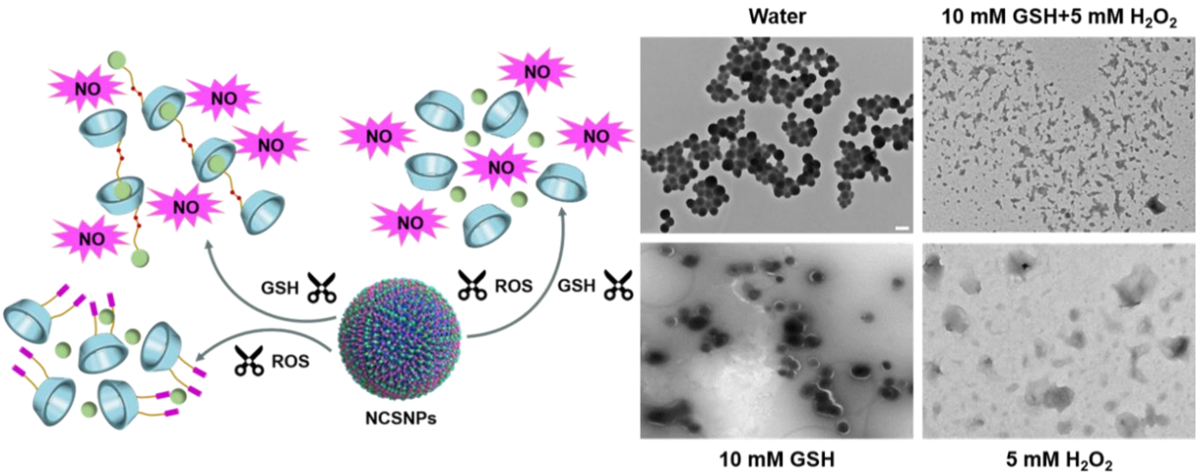

Figure S25 Schematic illustration of the processes involved in stimulus-response of NCSNPs (left). TEM image of NCSNPs after incubation with water, 10 mM GSH, 5 mM H_2O_2, and the mixture of GSH and H_2O_2 for 24 h. The scale bar is 200 nm (right).

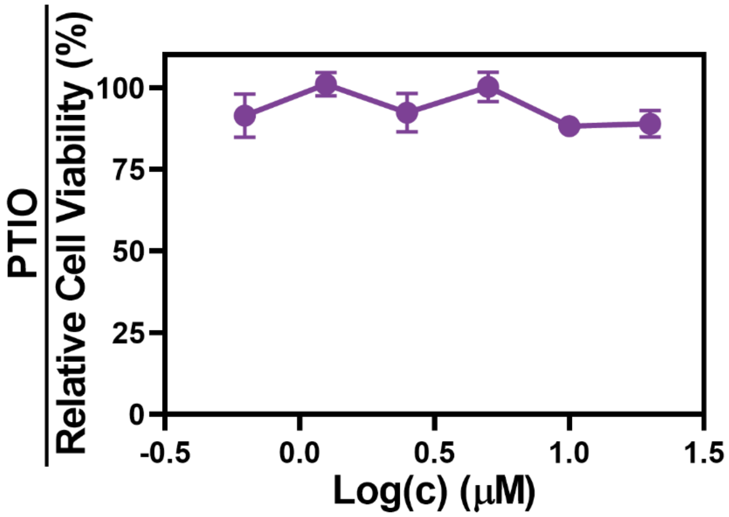

Figure S26 Viability curve of B16 cells after treatment with PTIO for 24 h.

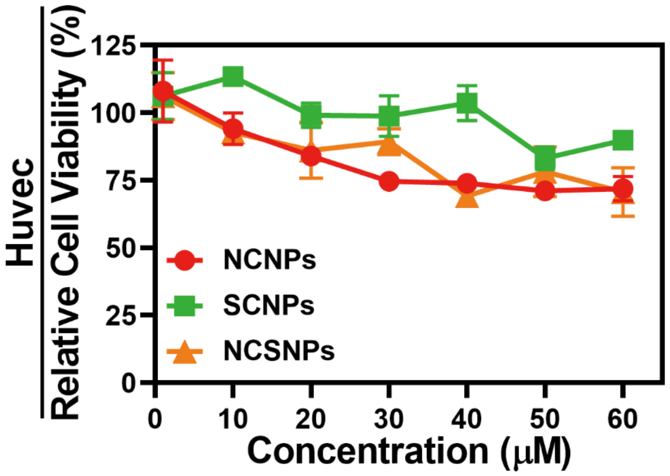

Figure S27 Viability curves of normal cells (Huvec) after treatment with NCSNPs for 24 h.

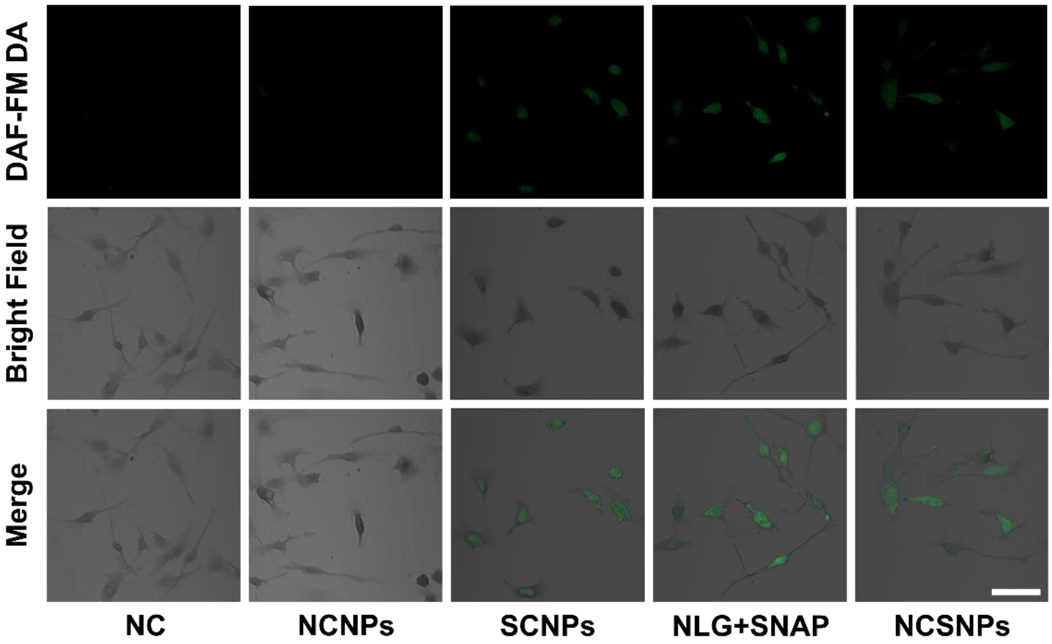

Figure S28 CLSM images of B16 cells incubated with NCNPs, SCNPs, NLG + SNAP, and NCSNPs. Green fluorescence comes from DAF. The scale bar is 20 μm.

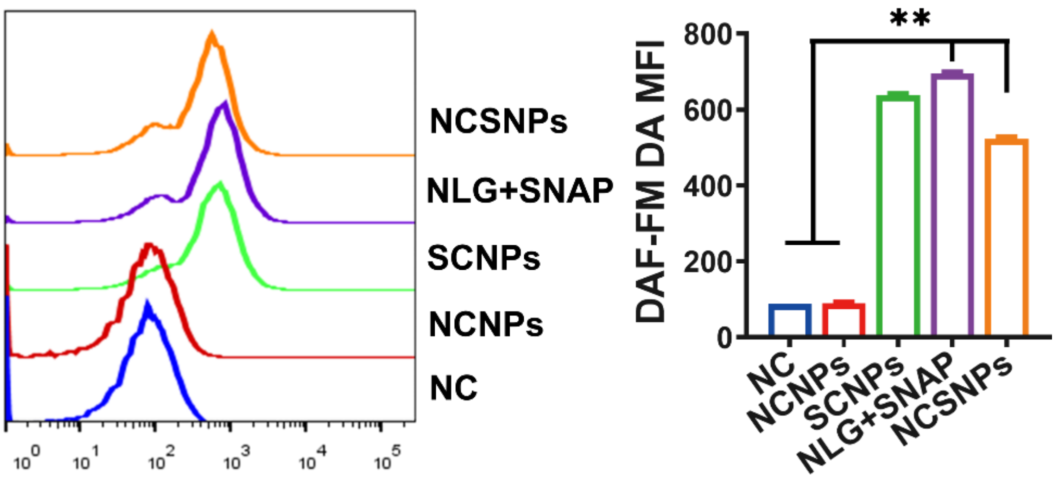

Figure S29 Intracellular Mean fluorescence intensity (MFI) changes of DAF-FM for the B16 cells treated with NCNPs, SCNPs, NLG + SNAP, and NCSNPs.

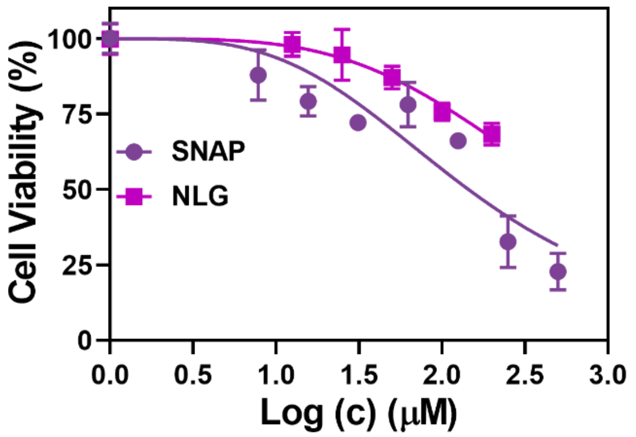

Figure S30 Viability curve of B16 cells after treatment with NLG and SNAP.

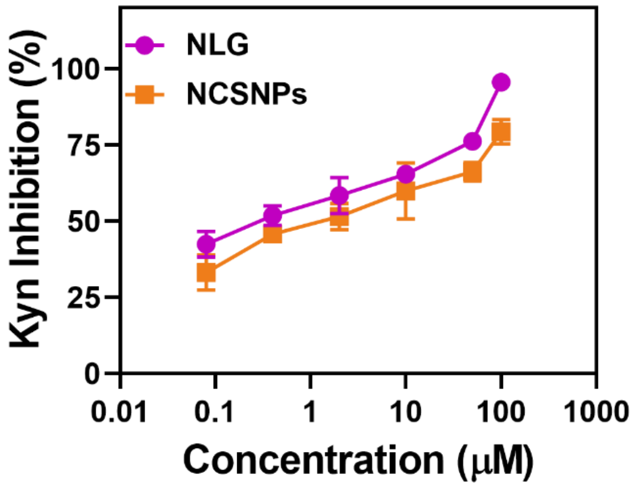

Figure S31 The B16 cells were treated with IFN-γ to induce IDO expression, and subsequently incubated with NLG and NCSNPs at different concentrations. The amount of Kyn was quantified by the absorbance at 480 nm.

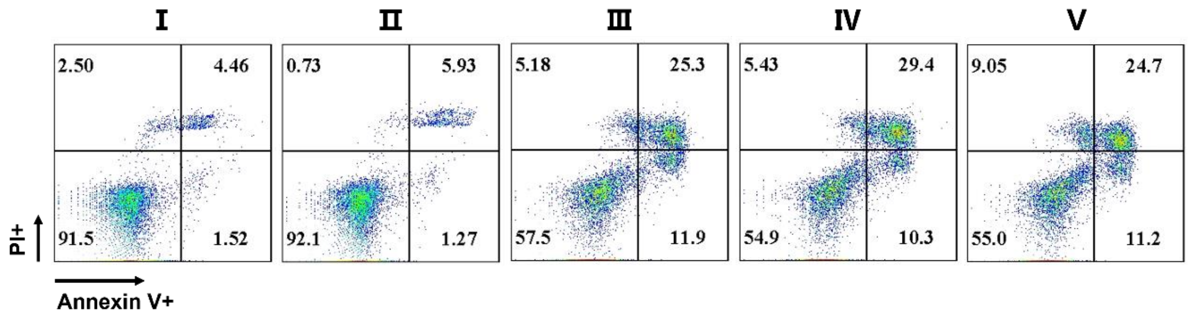

Figure S32 Annexin V-FITC/PI dual staining assays of B16 cells after incubation with various treatments for 24 h. I: control; II: NCNPs; III: SCNPs; IV: NLG+SNAP; V: NCSNPs.

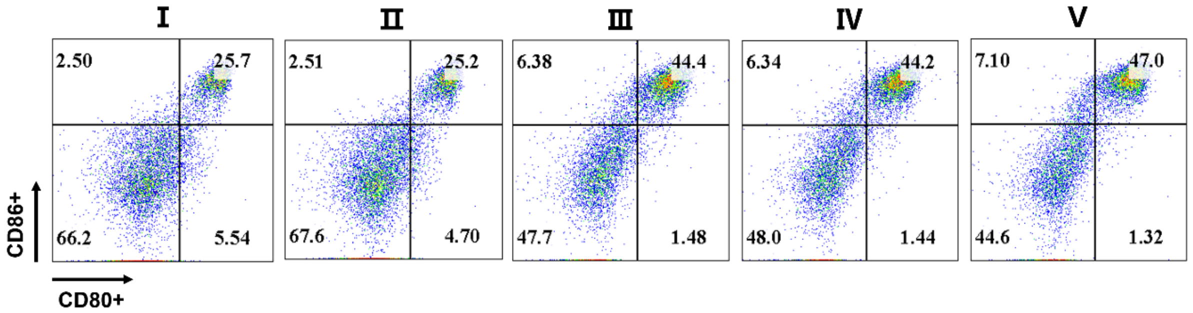

Figure S33 Representative results of the $CD80^+$ cells and $CD86^+$ cells in B16 cells after various treatments by flow cytometry. I: control; II: NCNPs; III: SCNPs; IV: NLG+SNAP; V: NCSNPs.

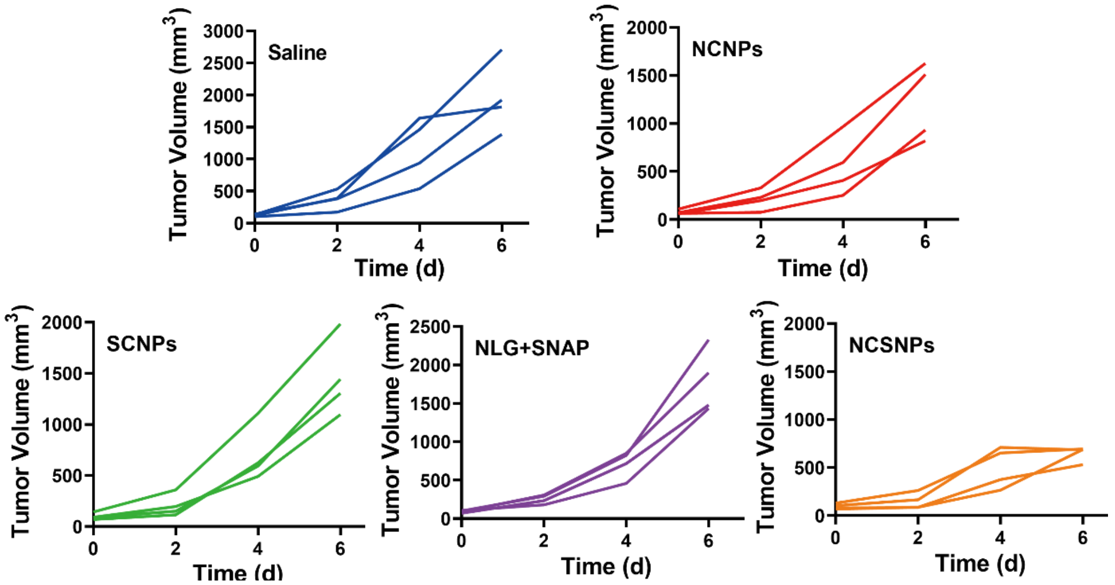

Figure S34 Individual B16 tumor growth curves after different treatments.

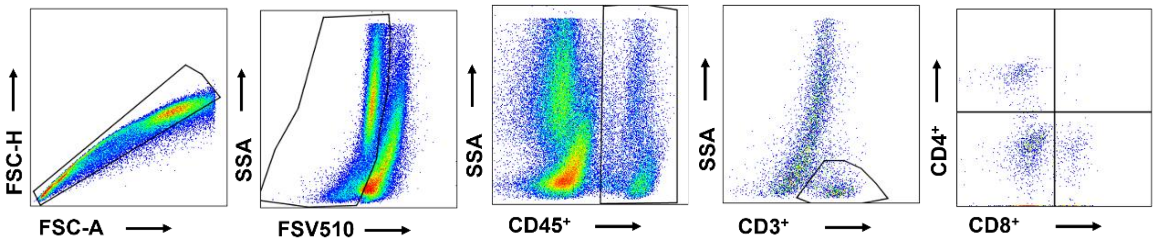

Figure S35 Flow cytometry gating strategy for the analysis of T cells in the tumor.

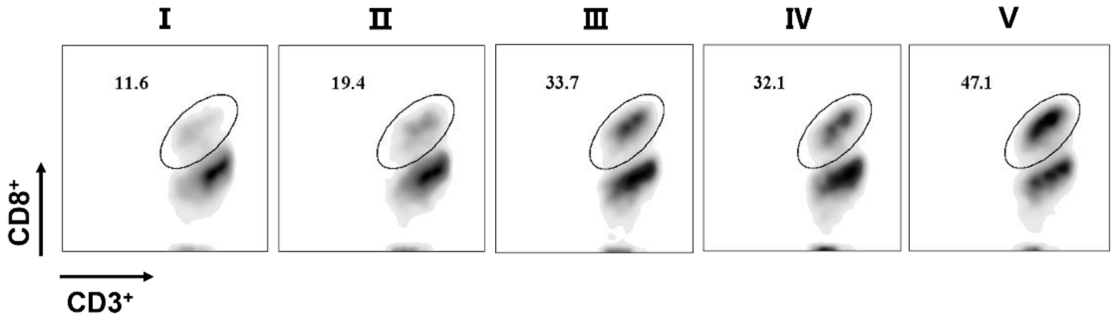

Figure S36 Representative flow cytometry plots of $CD3^+CD8^+$ cells gating on $CD3^+$ cells in tumors of B16 tumor-bearing mice after different treatments. I: saline; II: NCNPs; III: SCNPs; IV: NLG+SNAP; V: NCSNPs.

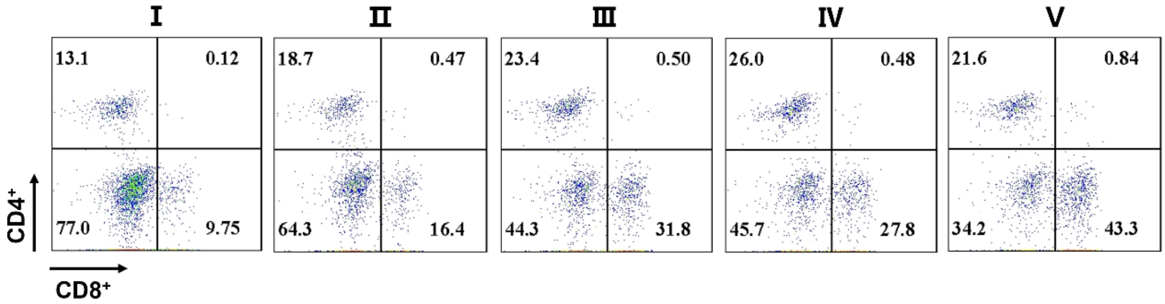

Figure S37 Representative results of the CD4$^+$ T cells and CD8$^+$ T cells in B16 cells after various treatments by flow cytometry. I: saline; II: NCNPs; III: SCNPs; IV: NLG+SNAP; V: NCSNPs.

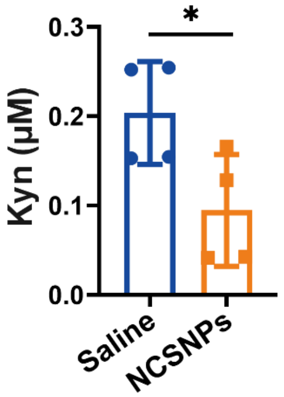

Figure S38 The intratumoral Kyn calculated from mice intravenously injected with saline and NCSNPs, respectively.

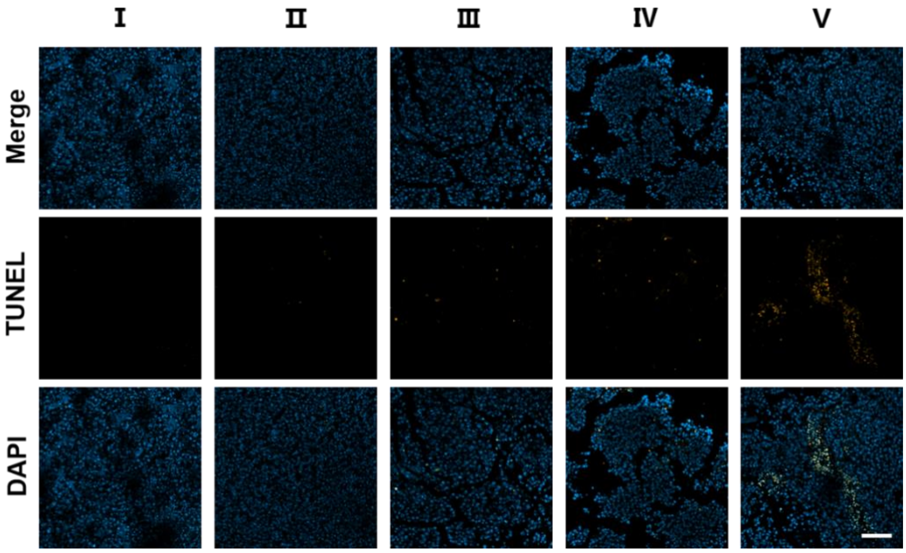

Figure S39 The TUNEL staining of tumor sections collected from mice intravenously injected with various treatments. I: saline; II: NCNPs; III: SCNPs; IV: NLG+SNAP; V: NCSNPs. The scale bar is 100 μm.

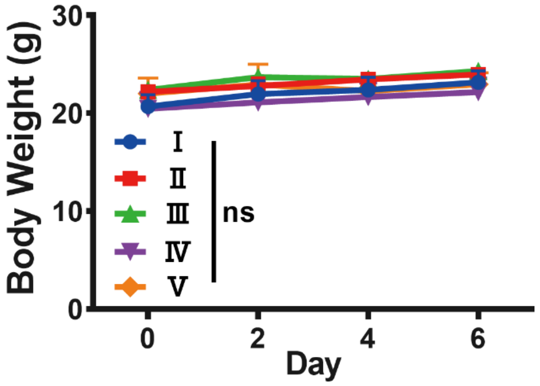

Figure S40 Body weight change of mice during the test. I: saline; II: NCNPs; III: SCNPs; IV: NLG+SNAP; V: NCSNPs.

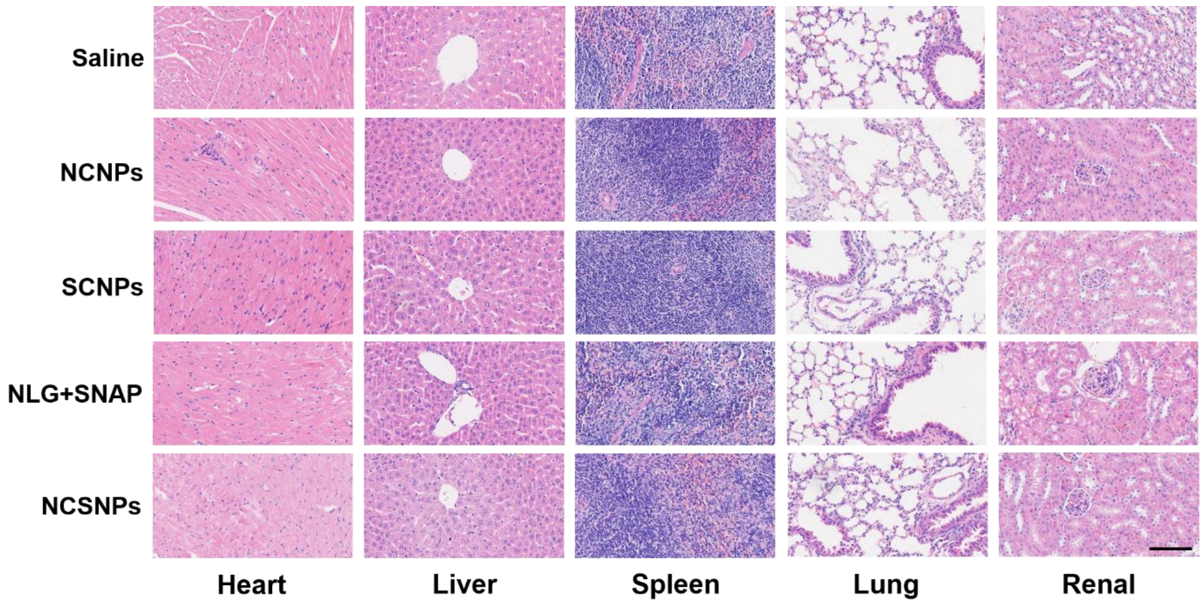

Figure S41 H&E staining of major organs (heart, liver, spleen, lung, kidney) harvested from B16 tumor-bearing mice by the end of different treatments. The scale bar is 100 μm.

6. References

1. Kang, Y.; Zhou, L.; Li, X.; Yuan, J., *J. Mater. Chem.*, **2011,** *21* (11), 3704-3710.
2. Yang, K.; Yu, G.; Yang, Z.; Yue, L.; Zhang, X.; Sun, C.; Wei, J.; Rao, L.; Chen, X.; Wang, R., *Angew. Chem. Int. Ed.*, **2021,** *60* (32), 17570-17578.
3. Zhou, F.; Feng, B.; Wang, T.; Wang, D.; Cui, Z.; Wang, S.; Ding, C.; Zhang, Z.; Liu, J.; Yu, H.; Li, Y., *Adv. Funct. Mater.*, **2017,** *27* (46), 1703674.
4. Yang, K.; Qi, S.; Yu, X.; Bai, B.; Zhang, X.; Mao, Z.; Huang, F.; Yu, G., *Angew. Chem. Int. Ed.*, **2022,** *61* (27), e202203786.
5. Yu, G.; Zhao, X.; Zhou, J.; Mao, Z.; Huang, X.; Wang, Z.; Hua, B.; Liu, Y.; Zhang, F.; He, Z.; Jacobson, O.; Gao, C.; Wang, W.; Yu, C.; Zhu, X.; Huang, F.; Chen, X., *J. Am. Chem. Soc.*, **2018,** *140* (25), 8005-8019.
6. Matin, A.; Streete, I. M.; Jamie, I. M.; Truscott, R. J. W.; Jamie, J. F., *Anal. Biochem.*, **2006,** *349* (1), 96-102.
